# A novel amniote model of epimorphic regeneration: the leopard gecko, *Eublepharis macularius*

**DOI:** 10.1186/1471-213X-11-50

**Published:** 2011-08-16

**Authors:** Katherine E McLean, Matthew K Vickaryous

**Affiliations:** 1 Department of Biomedical Sciences, University of Guelph, 50 Stone Road East, Guelph, ON, Canada; 2 Faculty of Medicine, University of Toronto, 1 King's College Circle, Toronto, ON, Canada

## Abstract

**Background:**

Epimorphic regeneration results in the restoration of lost tissues and structures from an aggregation of proliferating cells known as a blastema. Among amniotes the most striking example of epimorphic regeneration comes from tail regenerating lizards. Although tail regeneration is often studied in the context of ecological costs and benefits, details of the sequence of tissue-level events are lacking. Here we investigate the anatomical and histological events that characterize tail regeneration in the leopard gecko, *Eublepharis macularius*.

**Results:**

Tail structure and tissue composition were examined at multiple days following tail loss, revealing a conserved pattern of regeneration. Removal of the tail results in a consistent series of morphological and histological events. Tail loss is followed by a latent period of wound healing with no visible signs of regenerative outgrowth. During this latent period basal cells of the epidermis proliferate and gradually cover the wound. An additional aggregation of proliferating cells accumulates adjacent to the distal tip of the severed spinal cord marking the first appearance of the blastema. Continued growth of the blastema is matched by the initiation of angiogenesis, followed by the re-development of peripheral axons and the ependymal tube of the spinal cord. Skeletal tissue differentiation, corresponding with the expression of Sox9, and muscle re-development are delayed until tail outgrowth is well underway.

**Conclusions:**

We demonstrate that tail regeneration in lizards involves a highly conserved sequence of events permitting the establishment of a staging table. We show that tail loss is followed by a latent period of scar-free healing of the wound site, and that regeneration is blastema-mediated. We conclude that the major events of epimorphic regeneration are highly conserved across vertebrates and that a comparative approach is an invaluable biomedical tool for ongoing regenerative research.

## Background

Epimorphic regeneration is a post-traumatic morphogenetic event characterized by the aggregation of proliferating cells at the wound site [1]. Ultimately, this mass of proliferating cells--the regeneration blastema--differentiates to form new tissues that replace lost or damaged structures and organs [1]. To date, most research on naturally evolved epimorphic regeneration in vertebrates has focused on non-amniotes including teleosts (e.g., zebrafish) and urodeles (e.g., axolotls and newts) [2]. Although various amniotes (reptiles and mammals) are capable of regenerating multi-tissue structures, true epimorphic (i.e., blastema-mediated) regeneration is comparatively rare. One obvious exception is the tail of many lizards.

**Figure 1:**
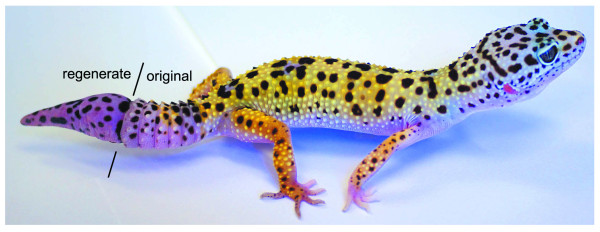
**The leopard gecko, *Eublepharis macularius***. *Eublepharis macularius*, the leopard gecko. Lateral view of an adult with a regenerate tail. **Lines **identify the proximal extent of the regenerate portion of the tail.

It is well understood that many lizards are able to voluntarily shed or autotomize their tail as a strategy to escape predation [3]. Tail autotomy is typically followed by tail regeneration. Beginning with the formation of a cellular aggregation (reportedly a blastema), these lizards are able to develop a replacement appendage that, at least superficially, resembles the original, complete with nerves, blood vessels and skeletal support. Although often explored in the context of ecological costs and benefits [4], less is known about the sequence of cellular- and tissue-level events of lizard tail regeneration. With this in mind we introduce the leopard gecko, *Eublepharis macularius*, as a laboratory-amenable model for the study of regeneration (Figure 1). *E. macularius *is a hardy, commercially bred lizard with a conservative morphology (five digits per limb, no trunk elongation) and well-established husbandry protocols [5,6]. Furthermore, the tail of *E. macularius *is able to autotomize and regenerate naturally (Figure 1).

As for all lizards, the original tail of *E. macularius *is a prominent and complex appendage composed of multiple tissue types including striated muscle, vasculature, adipose tissue, a bony vertebral column and a spinal cord. Unlike other lizards, geckos (including *E. macularius*) also retain the notochord into skeletal maturity. In most amniotes this rod-like organ degenerates during embryonic development (except for small remnants in the intervertebral discs, the nuclei pulposi) [7]. In geckos, the persistent notochord passes through the centra and intervertebral discs of the entire vertebral column. The tail also demonstrates a number of anatomical modifications that facilitate autotomy. These include intravertebral fracture planes, unmineralized gaps that pass transversely through most of the caudal (tail) vertebrae to permit controlled breakage of the skeleton, and arterial sphincters that contract to minimize blood loss when the tail is detached [3,8].

Among those lizards able to regenerate, the new tail is superficially similar to the original but not a perfect replica [3]. For example, the original axial skeleton consists of a series of bony vertebrae and persistent notochord, while in the regenerate tail these structures are replaced by a hollow cartilaginous cone [3,8,9]. In addition, the spinal cord of the original tail is incompletely restored during regeneration, limited to the ependymal cells surrounding the central canal (the ependymal tube), descending nerves tracts (encircling the ependymal tube) and meninges [10-12]. Externally, the scalation pattern of the regenerate tail also differs slightly from the original tail [10].

Here we describe the gross morphological and histological changes that occur throughout tail regeneration in *E. macularius*. Whereas previous investigations of lizard tail regeneration have described morphological changes in the context of absolute time, we establish a staging table that more accurately categorizes regenerative events. Staging provides a standardized method of comparing changes in morphology during both development and regeneration. For ectotherms (including reptiles and amphibians), variation in the rate of regeneration and morphogenesis is profoundly influenced by environmental factors (e.g., temperature, humidity), as well as the type and availability of nutrients, the chronological age of the organism and seasonal behaviours [3,12]. For example, tail regeneration in the gecko *Tarentola mauritanica *was observed to occur more rapidly when the animals were maintained at an ambient temperature of 35°C compared with those maintained at 28°C [13]. Hence the use of absolute time is inappropriate and staging provides the only reliable means of addressing these variables and conducting repeatable intra- and inter-specific comparisons [14,15]. Our data reveals that lizard tail regeneration follows a conserved sequence of morphological and histological events, including wound healing, blastema formation and differentiation, comparable with the events observed in other vertebrate regeneration models.

## Results

### Staging

To document regeneration we established a complete staging system of normal (i.e., otherwise unmanipulated) tail regeneration following autotomy, beginning with a detailed description of the original tail anatomy. The minimum timeframe to fully regenerate the tail (i.e., to achieve stage VII of a seven stage regeneration sequence) was 25 days at an ambient temperature of ~24°C (see Methods). Each stage of regeneration is based on one or more discrete and easy to recognize morphological features (Table 1). Morphological stages were then investigated using histology to further document the structural changes that characterize regeneration.

**Table 1:** Summary of key gross morphological and histological criteria used to define the stages of tail regeneration in *Eublepharis macularius*

Stage	Criteria
	→ autotomized vertebra exposed but no adjacent muscle or connective tissue retraction
I	→ spinal cord retracts within neural canal and is capped distally by a small blood clot
	→ integument begins to collapse around the wound site
	
	→ autotomized vertebra exposed and adjacent muscles and connective tissues retracted
	→ large clot caps entire wound surface
II	→ integument collapsed around wound site
	→ wound epithelium proliferates and begins to spread across autotomy surface deep to clot
	→ blastema appears between retracted spinal cord and overlying clot
	→ first appearance of multinucleated osteoclasts and chondroclasts adjacent to autotomized vertebra
	
	→ clot is lost and the complete wound epithelium is exposed
	→ wound epithelium continues to proliferate and thicken
III	→ apical epithelial cap forms
	→ blastema spreads beyond neural canal to cap distal end of the tail deep to wound epithelium
	→ angiogenesis begins within the blastema
	→ ependymal tube and axons from the dorsal root ganglia of the original tail begin to grow into blastema
	
IV	→ regenerating tail is dome-shaped and wider than long (length: diameter is less than 0.5)
	
	→ regenerating tail is an elevated dome and wider than long (length: diameter is greater than 0.5 but less than 1.0)
V early	→ earliest appearance of presumptive cartilaginous skeleton around the ependymal tube
	→ muscle tissue begins to differentiate
	
	→ regenerating tail is an elevated dome and wider than long (length: diameter is greater than 0.5 but less than 1.0)
V late	→ keratinization and the formation of scales begins within the epidemis
	→ first expression of Sox9 in cartilage cells of regenerating cartilage cone and original tail cartilage
	
	→ regenerating tail is a tapering cone approaching a length: width of 1.0
VI	→ first appearance of the differentiated dermis
	→ differentiation of cartilage, muscle and adipose tissue nearing completion
	
	→ regenerating tail is a tapering cone longer than wide (length: diameter is greater than 1.0)
VI	→ proximal portion of the regenerate tail is equal in diameter to the distal portion of original tail
	→ pigmentation begins

### Original Tail Anatomy

#### Gross Morphology and Histology

**Figure 2:**
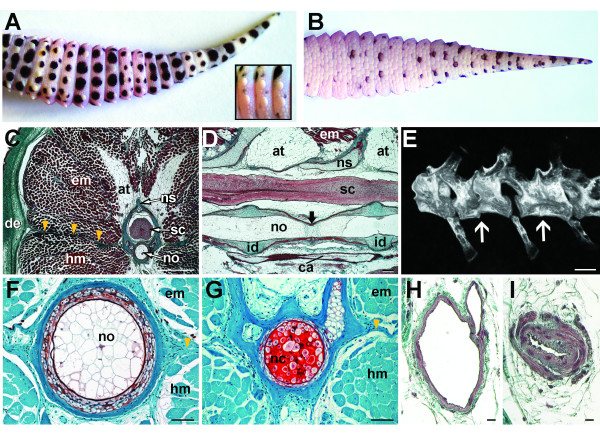
**Original anatomy of the gecko tail**. Original tail anatomy. *Eublepharis macularius*. **(A,B) **In dorsal **(A) **and ventral views **(B)**. Note the tubercles **(inset) **and the difference in pigmentation. **(C-D, F-I) **Serial sections (dorsal towards top of page). **(C) **Transverse section through distal third of the tail, stained with Masson's trichrome. The spinal cord (stained pink) is centrally positioned, enclosed within the neural canal of a vertebra (in this view stained green with red mottles). Surrounding the skeleton are bands of adipose tissue and skeletal muscle (stained red), enclosed by dermis (stained green) and epidermis (stained red). A longitudinal connective tissue septum radiates from the transverse process (**yellow arrows**). **(D) **Sagittal section through vertebral column, stained with Masson's trichrome. Ventral and parallel to the spinal cord is the notochord (in this view stained red with green mottles) and intervertebral disc (stained green). Ventral to the vertebral column is the caudal artery (stained red). The position of the intravertebral fracture plane indicated **(black arrow)**. **(E) **Micro-computed tomography reconstruction of tail (caudal) vertebrae. Position of the intravertebral fracture planes indicated **(white arrows)**. **(F,G) **Transverse sections through notochord in areas containing choroid tissue **(F) **and notochordal cartilage **(G)**, stained with Safranin O. **(H,I) **Transverse sections through caudal artery before **(H) **and at the caudal arterial sphincter **(I)**, stained with Masson's trichrome. at, adipose tissue; ca, caudal artery; de, dermis; em, epaxial musculature; hm, hypaxial musculature; id, intervertebral disc; nc, notochordal cartilage; no, notochord; ns, neural spine; sc; spinal cord. Scale bars: c, e = 500 μm; d = 100 μm; f = 50 μm; g = 20 μm; h,i = 10 μm; j-l = 20 μm.

The original tail resembles a tapering cone and represents approximately 41% of the total body length (Additional file 1). The pattern of scalation across the tail is dominated by large numbers of relatively small flattened, overlapping scales (Figures 2A and 2B). Along the dorsal and lateral surfaces this pavement of flattened scales is interrupted at regular intervals by large cone-like tubercles (Figure 2A). Close inspection reveals that adjacent, slightly enlarged scales surround each tubercle. On the ventral surface the flattened scales are imbricated (Figure 2B). Similar to the rest of the body the tail demonstrates a characteristic pattern of countershading, with a white ventral surface and pigmented dorsal and lateral surfaces. The typical pigmentation pattern includes a background colour ranging from yellow to orange or white with variable numbers of irregularly shaped dark brown to black spots and stripes (Figures 2A and 2B).

#### Histology

In transverse section the tail is circular to ovoid, with a centrally located skeletal system. In this plane the tail is subdivided into quadrants by longitudinal connective tissue septa radiating from the neural spine (dorsally), chevron (ventrally) and transverse processes (laterally). Each dorsal (epaxial) and ventral (hypaxial) quadrant includes varying amounts of adipose, connective tissue and musculature (Figure 2C).

The original skeletal system includes two distinct axial elements: a series of caudal vertebrae (and associated chevrons; Figures 2C-2E) and a persistent notochord (Figures 2C, D, F and 2G) (Additional file 2). As for other geckos, in *E. macularius *the notochord is retained as a rod-like structure that passes through each vertebral centra, beginning in the cervical series and terminating at the distal tip of the tail. The notochord is an avascular, aneurogenic structure consisting of a fibrous connective tissue and elastin sheath, surrounding two alternating tissues types: chordoid and chondroid tissue. Chordoid tissue is dominated by large vacuolated cells (chordocytes) with small nuclei and limited extracellular matrix (Figure 2F). At regular intervals chordoid tissue is interrupted by segments of cartilage-like chondroid tissue known as notochordal cartilage (Figure 2G). Compared with chordoid tissue, notochordal cartilage is rich in extracellular matrix with smaller cells that lack vacuoles. Both chordoid tissue and notochordal cartilage stain positive for glycosaminoglycans (Figures 2F and 2G). Although the alternating arrangement of chordoid tissue and notochordal cartilage is constant along the entire length of the notochord, in the tail the distribution of chondroid tissue corresponds with the location of intravertebral fracture planes (see below).

Each caudal vertebra consists of a centrum, neural arch and spine, bilateral transverse processes, and a freely articulating chevron. As for other geckos, most of the centra are strongly amphicoelous with a conspicuous canal for passage of the notochord. In longitudinal section this canal has an hourglass-shape that narrows at mid-centrum (Figure 2D). These narrow segments correspond with the presence of notochordal cartilage (Figure 2D). The remainder of the notochordal canal is occupied by chordoid (vacuolated cell-rich) tissue.

With the exception of the cranial-most and the caudal-most caudal vertebrae, each caudal centrum (and corresponding neural arch) is transversely partitioned by an autotomy plane, a near vertical unmineralized gap (Figure 2E). The cranial and caudal portions of each vertebra are united by a transversely radiating extension of fibrous connective tissue (the fracture plane - see below) and the notochord. Autotomy planes facilitate voluntary tail loss by permitting the vertebra to be split in a specific location.

The spinal cord passes continuously along the length of the tail dorsal to the centra (Figure 2D; see Additional file 2) and is flanked dorsolaterally at regular intervals by dorsal root ganglia and spinal nerves. In section, the spinal cord is composed of a near central ependymal tube (enclosing the central canal), dorsal and ventral gray columns, and white matter (Figure 2C). At the level of light microscopy the spinal cord does not demonstrate any obvious adaptations for autotomy.

Although various blood vessels pass through the tail, this investigation will only focus on two. Ventral to each centrum and partially enclosed by the chevrons is the largest arterial vessel of the tail, the caudal artery. For most of its length the caudal artery has relatively thin walls and a large luminal diameter (Figure 2H). However, at regular intervals immediately proximal to each fracture plane, the caudal artery develops thick smooth muscle sphincters (Figure 2I). Following autotomy, these sphincters are reported to constrict thus preventing blood loss [3]. The second vessel of interest is the small diameter spinal artery passing subadjacent to the spinal cord. The spinal artery lacks smooth muscle sphincters but appears to play an important role in clot formation immediately following autotomy (see below).

Surrounding the vertebral column are four longitudinal bands (paired epaxial and hypaxial) of perivertebral adipose tissue and supra-adjacent musculature. Each adipose tissue band contains numerous small blood vessels and nerves, and is surrounded by a fibrous connective tissue sheath. The adipose tissue bands diminish in thickness caudally and are surrounded in turn by segmental bands of axial musculature (Figure 2C). Both the adipose tissue bands and axial musculature demonstrate a complex arrangement of myomeric segmentation, resulting in a prominent series of W-shaped zigzag interdigitations (Additional file 3). The transverse connective tissue septa defining these myomeric segments contribute to the soft tissue extensions of the fracture planes (see below).

The integument of the original tail is readily divisible into a superficial epidermis and a deeper dermis. As for other squamates, the epidermis is stratified into 4-7 layers and is heavily keratinized. The dermis consists of a stratum superficiale, composed of loose, irregular connective tissue, and a stratum compactum, composed of large, more densely organized connective tissue. Subadjcent to the dermis is a variably thick hypodermis, rich in adipose and loose connective tissue.

Each fracture plane is a transversely oriented bilayer of fibrous connective tissue penetrated by vasculature, lymphatics, and components of the nervous system. Beginning at the vertebra, the fracture plane is continuous with the fibrous portion of the periosteum at the unmineralized gap located in the mid-centrum region. The bilayer then blends with the transverse septa defining each segmented band of adipose tissue and axial musculature. At the dermis the fracture plane no longer resembles a bilayer, although it remains identifiable as a conspicuous localization of denser fibrous tissue. The fracture plane does not pass through the epidermis.

**Figure 3:**
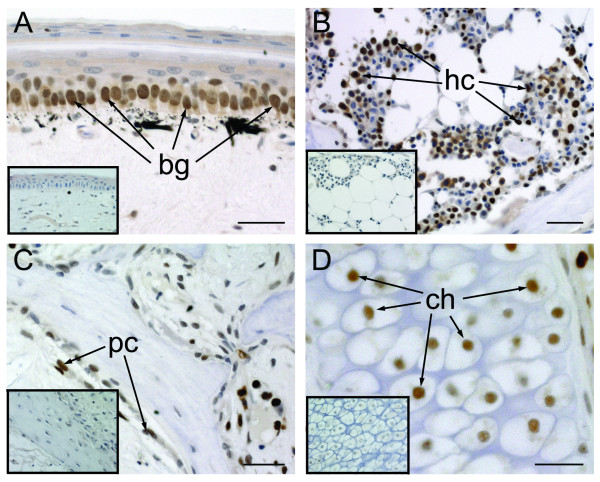
**Cell proliferation in the original tail**. Original tail tissues. *Eublepharis macularius*. **(A-D) **Longitudinal sections immunostained with PCNA (brown) and counterstained with hematoxylin (blue). Negative controls of comparable sections (omitting the primary antibody) included as inset images. **(A) **Basal germinative cells of the epidermis, **(B) **hematopoietic cells, **(C) **periosteal cells, and **(D) **chondrocytes. bg, basal germinative cell; ch, chondrocyte; hc, hematopoietic cell; pc, periosteal cell.

To identify proliferating cells we used proliferating cell nuclear antigen (PCNA) immunohistochemistry. PCNA (= cyclin) is a nuclear protein required for chromosomal DNA replication and is commonly used as a marker of the synthesis (S) phase of the cell cycle [16,17]. PCNA positive cells were observed in several locations throughout the original tail, including basal germinative cells of the epidermis, hematopoietic cells of the bone marrow and periosteal cells surrounding the vertebrae (Figures 3A-3C). Additionally, isolated PCNA positive cells were seen in among the chondroprogenitor cells associated with cartilage of the vertebral column (Figure 3D).

To identify early evidence of cartilage formation we used Sox9 immunohistochemistry. Sox9 is a transcription factor involved in the regulation of chondrogenesis and chondrocyte differentiation [18-20]. There were no Sox9 positive cells in the original tail (results not shown). Sox9 immunopositive cells were not detected in regenerating lizard tails until late Stage V.

### Stage I (0 - 24 hours post-autotomy)

#### Overview

**Figure 4:**
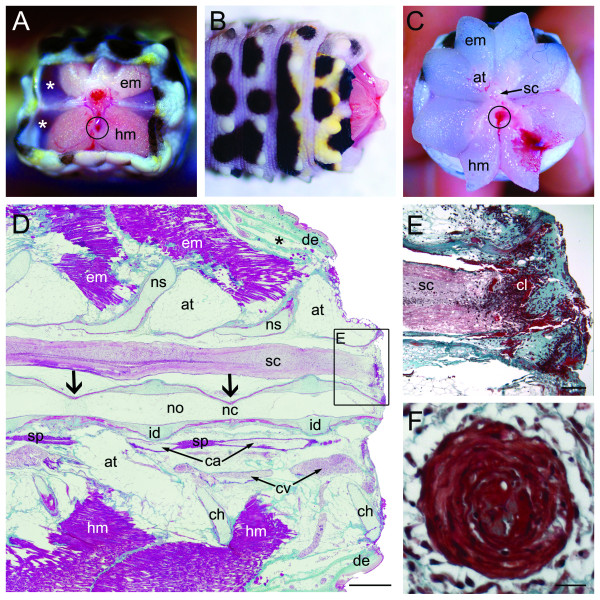
**Stage I of tail regeneration**. Stage I of tail regeneration. *Eublepharis macularius*. **(A,B) **Gross morphology of the autotomy surface in caudal **(A) **and dorsal view **(B)**. Integument begins to collapse/retract and various skeletal and non-skeletal tissues are observed to protrude. Recesses for projecting muscle processes from the autotomized tail are indicated **(white asterisks)**. Note the caudal artery **(circle)**. **(C) **Gross morphology of the autotomized portion of the tail in cranial view. Position of the caudal artery indicated (**circle**). Note the star-like pattern formed by projecting muscle processes. **(D-F) **Serial sections stained with Masson's trichrome (dorsal towards top of page). **(D) **Sagittal section of tail, (autotomy surface to right). Collapsed recess for projecting muscle process indicated **(black asterisk)**. The spinal cord (stained pink) is centrally positioned. Ventral and parallel to the spinal cord is the notochord (in this view stained red with green mottles) and intervertebral disc (stained green). Surrounding the skeletal are bands of adipose tissue (unstained) and skeletal muscle (stained red). Position of intravertebral fracture planes indicated (**black arrows**). **(E) **Closer view of the region identified in panel **(D) **taken from a different section. Note the developing clot (stained red and green) distal to the torn and retracted spinal cord (autotomy surface to right). **(F) **Transverse section through contracted sphincter of the caudal artery. at, adipose tissue; ca, caudal artery; cl, clot; ch, chevron; cv, caudal vein; de, dermis; em, epaxial musculature; hm, hypaxial musculature; no, notochord; nc, notochordal cartilage; ns, neural spine; sc, spinal cord; sp, arterial sphincter. Scale bars: d = 500 μm; e = 100 μm; f = 20 μm.

Immediately following tail loss, the autotomy surface resembles an open wound with various tissues exposed, including the proximal portion of the autotomized vertebra, spinal cord, adipose tissue, musculature, and dermis (Figure 4A). This stage is characterized by minimal blood loss from the caudal artery, the formation of a small clot located distally adjacent to the spinal cord and collapse of the integument surrounding the wound site to reduce the overall size of the wound (Figures 4A and 4B). The autotomized portion of the tail undulates violently for 5-12 minutes following detachment. During this time blood is observed to leak from the ruptured caudal artery and tongue-shaped processes of epaxial and hypaxial musculature protrude from the fractured surface (Figure 4C). These muscular processes correspond to recesses within the original tail stump.

#### Histology

The centrum, neural arch, neural spine, transverse processes and chevron are partially exposed and observed to project from the autotomy surface (Figures 4A and 4B). Serial histology confirms that the vertebra has split along the fracture plane at the unmineralized gap (Figure 4D). The notochord is also split at a location corresponding to a segment of notochordal cartilage. Centrally, the spinal cord appears to have been torn resulting in a frilled margin along the autotomy surface. Almost immediately after tail loss the spinal cord retracts from the autotomy surface into the neural canal, after which a blood clot caps the distal end (Figure 4E). This clot forms as a result of blood loss from the subadjacent spinal artery. Near the autotomy surface the smooth muscle sphincter of the caudal artery has contracted and appears tightly closed (Figure 4F).

Perivertebral adipose tissue and axial musculature are exposed along the autotomy surface, lined by a monolayer of the fibrous fracture plane. Immediately following tail loss the autotomy plane is characterized by four pairs of deep recesses corresponding to the interdigitating arrangement of the musculature. These recesses rapidly disappear as the integument collapses around the wound site (Figure 4D).

Cell proliferation remains largely restricted to the basal layer of the epithelium, bone marrow and periosteum. No PCNA positive cells were otherwise associated with the autotomy surface.

### Stage II (18 hours - 8 days post-autotomy)

#### Overview

**Figure 5:**
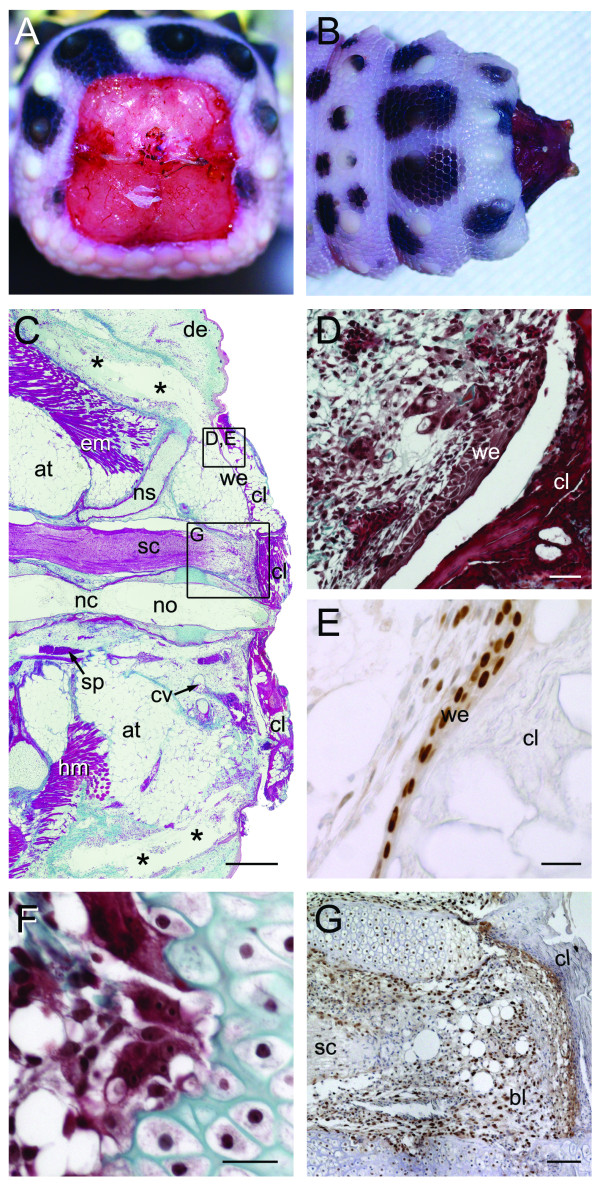
**Stage II of tail regeneration**. Stage II of tail regeneration. *Eublepharis macularius*. **(A,B) **Gross morphology of autotomy surface in caudal **(A) **and dorsal view **(B)**. Non-skeletal tissue has retracted from the autotomy surface further exposing the fractured vertebra. Serial sections stained with Masson's trichrome **(C,D,F) **or immunostained with PCNA **(E,G) **(dorsal towards top of page, autotomy surface to right). **(C) **Sagittal section of tail. The spinal cord (stained pink) is centrally positioned. Ventral and parallel to the spinal cord is the notochord (in this view stained red with green mottles). The centrally positioned skeleton is surrounded by bands of adipose tissue (unstained) and skeletal muscle (stained red). Note the recesses for projecting muscle processes from the autotomized tail (**black asterisks**). **(D,E) **Closer views of the region identified in panel **(C) **taken from different sections. Note the wound epithelium developing deep to the clot. Most of wound epithelium cells are positive for PCNA (immunostained brown) indicating they are proliferating **(E)**. **(F) **Closer view of a multinucleated chondroclast and associated Howship's lacuna carved into cartilage (stained green). **(G) **Longitudinal sections immunostained for PCNA (brown) and counterstained with hematoxylin (blue) identifies proliferating cells in the early blastema and some of the surrounding tissues **(G)**. at, adipose tissue; bl, blastema; cl, clot; cv, caudal vein; de, dermis; nc, notochordal cartilage; no, notochord; ns, neural spine; sc, spinal cord; sp, arterial sphincter; we, wound epithelium. Scale bars: c = 500 μm; d-f = 20 μm; g = 50 μm.

Stage II is characterized by retraction of the exposed soft tissues at the autotomy surface (e.g., adipose tissue, axial musculature) resulting in a more prominent protrusion of the vertebral remnant (Figures 5A and 5B). Further collapse and sealing of the original integument to partially enclose the autotomy surface, and the formation of an exudate clot that completely spans the open wound, is also observed.

#### Histology

The exudate clot is composed mostly of serum and tissue fluid, erythrocytes, dead cells and tissue debris. It is thickest and most densely organized distal to the spinal cord (Figure 5C). Deep to the exudate clot, cells from the adjacent epidermis are beginning to centripetally span across the autotomy surface (Figure 5D). This newly formed covering is termed the wound epithelium. Cells of the wound epithelium are PCNA positive (Figure 5E) and, at this stage, form a layer 1-4 cells thick. Matching with the onset of re-epithelialization of the wound site, large numbers of multinucleated osteoclasts and chondroclasts are observed associated with the protruding vertebral remnant (Figure 5F).

The spinal cord remains retracted from the wound surface. Distal to the spinal cord but deep to the clot is an aggregation of uniform PCNA positive cells (Figure 5G). These cells mark the earliest appearance of the blastema. Within the original tail the number of cell types immunopositive for PCNA has increased to include cells of the epidermis, dermis, adipose tissue and ependymal tube of the spinal cord (Additional files 4A and 4B).

### Stage III (4 - 8 days post-autotomy)

#### Overview

**Figure 6:**
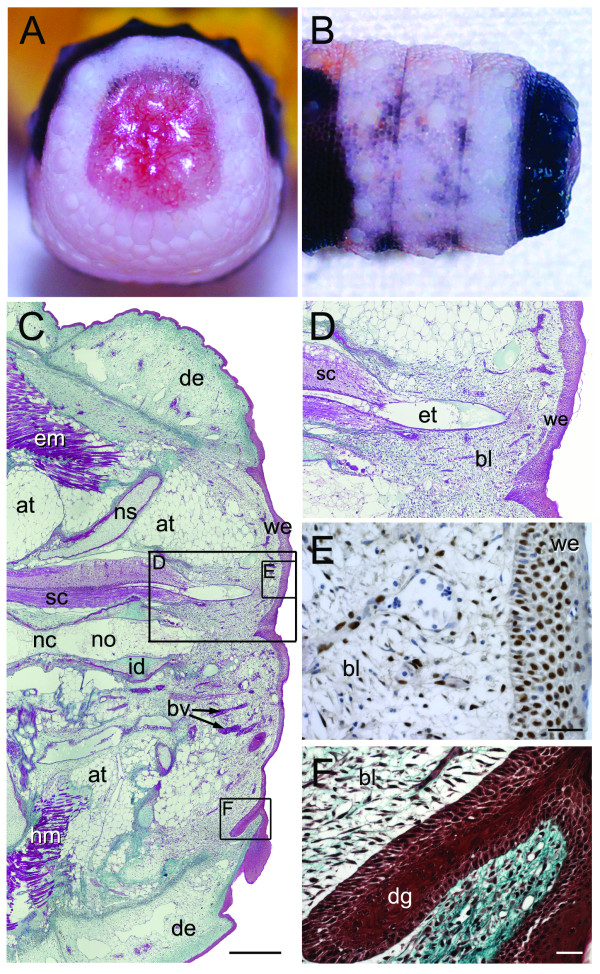
**Stage III of tail regeneration**. Stage III of tail regeneration. *Eublepharis macularius*. **(A,B) **Gross morphology of autotomy surface in caudal (**A**) and dorsal view **(B)**. The clot with protruding vertebral remnant is dropped and the fully formed wound epithelium is exposed. **(C-F) **Serial sections (dorsal towards the top of page, autotomy surface to the right) stained with Masson's trichrome **(C,E)**, immunostained with PCNA **(D) **or stained with hematoxylin and eosin **(F)**. **(C) **Sagittal section with the developing blastema (right side of image) beginning to emerge from the original tail. In the original tail the spinal cord (stained pink) is centrally positioned. It gives rise to the ependymal tube passing into the blastema (stained pink). Ventral and parallel to the spinal cord is the notochord. The notochord abruptly ends at the blastema. The blastema is capped by a thick wound epithelium (stained red). **(D) **Closer view of the region identified in panel **(C) **taken from the same section showing the regenerating ependymal tube invading the blastema. **(E) **Closer view of the region identified in panel **(C) **taken from a different section. Blastema and wound epithelium immunostained for PCNA (brown), to identify proliferating cells, and counterstained with hematoxylin (blue). **(F) **Closer view of the region identified in panel **(C) **taken from a different section. Note the prominent epidermal downgrowth (stained red). at, adipose tissue; bl, blastema; bv, blood vessels; dg, epidermal downgrowth; em, epaxial muscle; et, ependymal tube; hm, hypaxial muscle; id, intervertebral disc; no, notochord; sc, spinal cord; we, wound epithelium. Scale bars: c = 500 μm; d-f = 20 μm.

Stage III is characterized by loss of the exudate clot and exposure of the newly formed wound epithelium (Figure 6A). During this stage the autotomy surface is relatively flat with little evidence of outgrowth, and the once protruding vertebral remnant is no longer present (Figures 6A and 6B). Early during stage III newly developing blood vessels forming deep to the wound epithelium can be readily observed in caudal view (Figure 6A). By the end of stage III the wound epithelium has thickened and blood vessels are no longer obvious macroscopically.

#### Histology

Re-epithelialization is completed, with the wound epithelium spanning the entire autotomy surface (Figure 6C). During this stage the wound epithelium becomes increasingly thickened and stratified, beginning 3-5 cell layers thick and gradually increasing to 7-12 cell layers (Figure 6D). This thickening is most conspicuous at the apex of the regenerating tail and has been referred to as an apical epithelial cap (AEC) [10]. At the interface with the original epidermis the newly developing wound epithelium invades the collapsed recesses formerly occupied by tongue-shaped muscle processes. These deeply involuting structures are termed epidermal downgrowths (Figure 6E) and are rich in PCNA positive epithelial cells. In serial section epidermal downgrowths are observed to frame the blastema.

Prior to stage III, the distal-most vertebra consists of a remnant representing the cranial half of the original element. Following the action of osteoclasts and chondroclasts and ablation of the exudate clot, the wound epithelium is able to seal off the wound site. Ablation of the protruding portion of the distal-most vertebra further diminishes the length of this element to approximately one quarter its original size (Figure 6C). This quartered remnant no longer projects past the wound site, making it level with the original spinal cord, adipose tissue and musculature.

The blastema is well-established at this stage, distal to the original tissues and deep to the wound epithelium. The appearance of capillaries within the otherwise uniform population of PCNA positive cells marks the first evidence of new tissue formation (Figure 6F). Stage III is also characterized by the initiation of outgrowth from the original nervous system. These events begin as ependymal cells lining the central canal of the spinal cord begin to proliferate and peripheral axons from dorsal root ganglia in the original tail are observed to penetrate into the blastema.

Similar to stage II, stage III includes PCNA positive cells throughout the original tail tissues including the epidermis, dermis, adipose tissue and ependymal tube of the spinal cord (Additional files 4C and 4D). In the regenerating portion of the tail, cells within most layers of the wound epithelium and most cells within the blastema are PCNA positive (Figure 6D).

### Stage IV (8 - 15 days post-autotomy)

#### Overview

**Figure 7:**
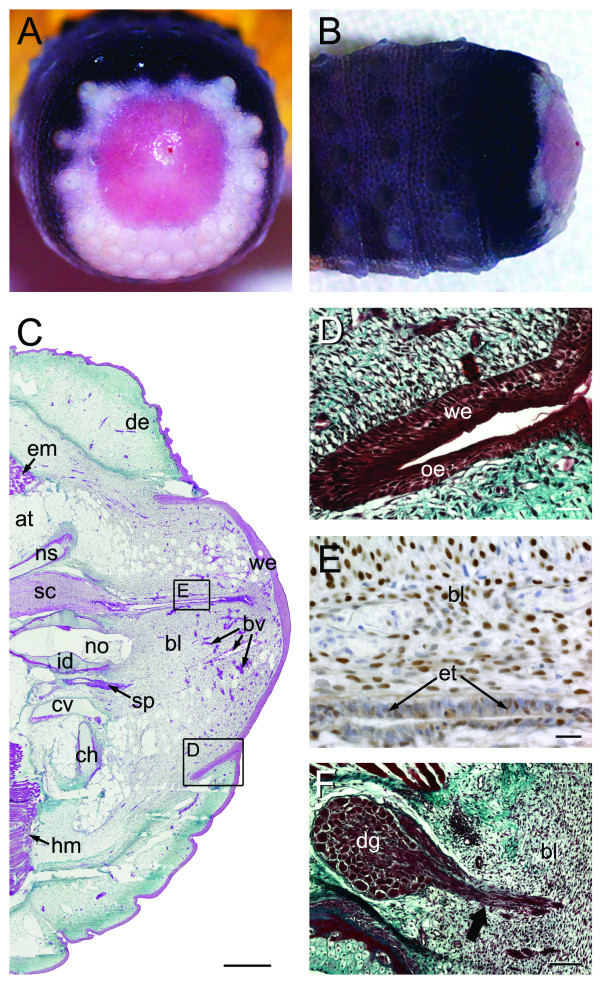
**Stage IV of tail regeneration**. Stage IV of tail regeneration. *Eublepharis macularius*. **(A,B) **Gross morphology of the regenerating tail in caudal **(A) **and dorsal view **(B)**. Regenerating tail is dome-shaped with a length: diameter less than 0.5. Serial sections stained with Masson's trichrome **(C,D,F) **or immunostained for PCNA **(E) **(dorsal towards top of page, autotomy surface to right). **(C) **Sagittal section of tail with the developing dome-shaped blastema (right side of image). The spinal cord (stained pink) is centrally positioned and continues into the blastema as the ependymal tube (stained pink). Ventral and parallel to the spinal cord is the notochord. The notochord abruptly ends at the blastema. The blastema is capped by a thick wound epithelium (stained red). **(D) **Closer view of the region identified in panel **(C) **taken from a different section. The interface of original epidermis and wound epithelium occurs at the epidermal downgrowth. **(E) **Closer view of the region identified in panel **(C) **taken from a different section. Ependymal tube and surrounding blastema is immunostained for PCNA (brown), to identify proliferating cells, and counterstained with hematoxylin (blue). **(F) **Closer view of a dorsal root ganglion with regenerating axons (strained red) passing into the blastema **(black arrow)**. at, adipose tissue; bl, blastema; bv, blood vessel; ch, chevron; cv, caudal vein; dg, epidermal downgrowth; de, dermis; em, epaxial muscle; et, ependymal tube; id, intervertebral disc; no, notochord; ns, neural spine; oe, original epithelium; sc, spinal cord; sp, arterial sphincter; we, wound epithelium. Scale bars: c = 500 μm; d,e = 20 μm; f = 100 μm.

Stage IV is the first stage in which there is well-defined outgrowth of the regenerating tail. More specifically, the regenerating portion of the tail is dome shaped in dorsal, ventral and lateral views (Figures 7A and 7B). Characteristically, the regenerate tail diameter is greater than regenerate tail length (the length to diameter ratio is less than 0.5). During this stage the regenerating tail is unpigmentated and appears light pink in colour (Figure 7A).

#### Histology

The wound epithelium has become thicker than the adjacent original epidermis (7-12 cell layers thick compared to the 4-7 for the original epidermis; Figures 7C and 7D) and epidermal downgrowths remain prominent. The blastema of stage IV tails continues to be dominated by PCNA positive blastema cells (Figure 7E).

By stage IV the ependymal tube has penetrated deep into the blastema, terminating just proximal to the wound epithelium (Figures 7C and 7E). Distally, the ependymal tube forms a blind-ended and slightly dilated structure known as the ependymal ampulla.

Peripheral axons from spinal nerves of the original tail continue to invade the blastema (Figure 7F) along with numerous capillaries. This stage also marks the initiation of regenerative myogenesis, starting with the aggregation of mononuclear myoblasts. Condensations of myoblasts first appear within the proximal portion of the blastema and thereafter demonstrate a distinct proximal to distal differentiation gradient. Matching the origination of these condensations, myoblasts closest to the original tail are the first to become elongate and fusiform in shape, and the first to demonstrate evidence of cell alignment. Centripetal to the developing muscles is a zone of slightly more densely organized blastema cells - the presumptive cartilaginous cone.

Compared with stage III, the number of PCNA positive cells in the original portion of stage IV tails has diminished (Additional files 4E and 4F). Where present, these cells are generally restricted to the basal layer of the epithelium, bone marrow and periosteum. Limited numbers of PCNA positive cells are found within the original dermis and spinal cord (including ependymal tube). In contrast, the regenerate portion of the tail continues to demonstrate large numbers of PCNA positive cells, including most cells of the wound epithelium and blastema, as well as endothelial cells in the newly formed capillaries.

### Stage V (12 - 26 days post-autotomy)

#### Overview

**Figure 8:**
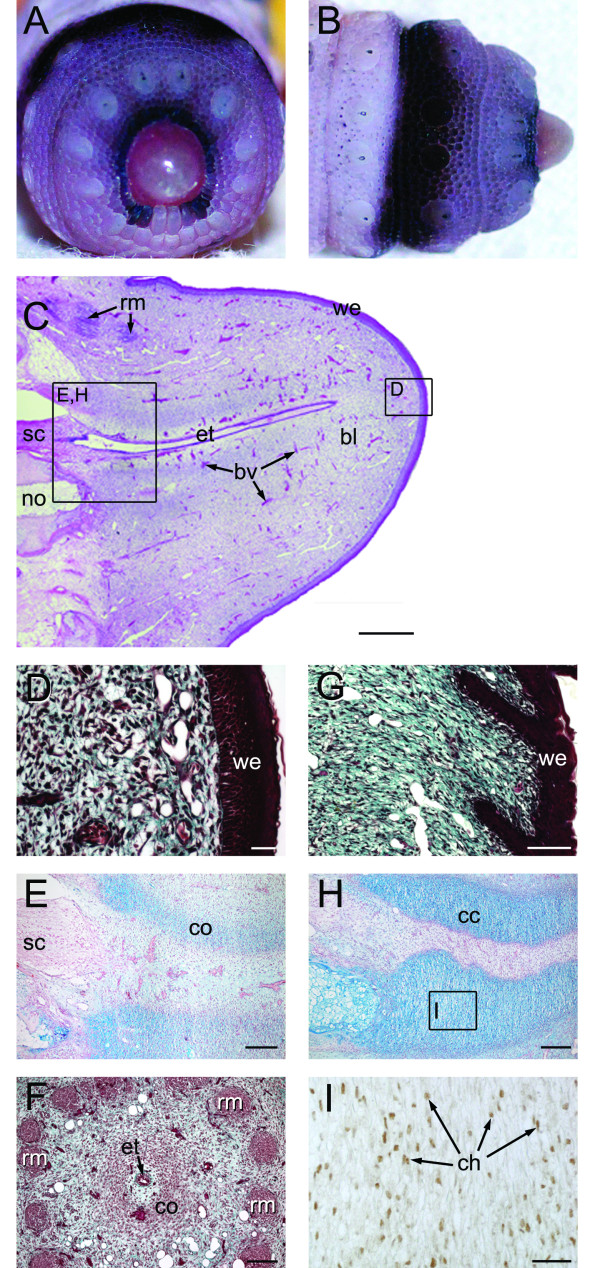
**Stage V of tail regeneration**. Stage V of tail regeneration. *Eublepharis macularius*. **(A,B) **Gross morphology of the regenerating tail in caudal **(A) **and dorsal view **(B)**. The regenerating tail is an elevated dome with a length: diameter between 0.5 and 1.0. Serial sections (dorsal towards the top) stained with hematoxylin and eosin **(C)**, Masson's trichrome **(D,G,F)**, Alcian blue **(E,H) **or immunostained with Sox9 **(I)**. **(C-F) **Early stage V. **(C) **Sagittal section of tail with the developing blastema (right side of image) shaped like an elevated dome. **(D) **Closer view of the region identified in panel **(C) **taken from a different section. Note the wound epithelium and underlying blastema. **(E) **Closer view of the region identified in panel **(C) **taken from a different section. In this image the precartilaginous mesenchymal condensation is visualized by weakly positive staining for Alcian blue. **(F) **Transverse section through the regenerate tail depicting early muscle formation (stained red) and precartilaginous mesenchymal condensation surrounding the ependymal tube. **(G-I) **Late stage V. **(G) **Closer view of epidermal ingrowths, the first evidence of scalation. **(H) **Parasagittal section through developing cartilaginous cone visualized by strong, positive staining for Alcian blue. **(I) **Closer view of cartilage cone immunostained for Sox9 (brown), to identity differentiating chondrocytes, and counterstained with hematoxylin (blue). bl, blastema; bv, blood vessel; cc, cartilage cone; co, mesenchymal condensation; em, epaxial muscle; et, ependymal tube; hm, hypaxial muscle; no, notochord; rm, regenerated muscle; sc, spinal cord; we, wound epithelium. Scale bars: c = 20 μm; d = 500 μm; e-g = 100 μm.

At stage V the distal tip of the regenerating tail resembles an elevated dome with a regenerate tail length to diameter ratio greater than 0.5 but less than 1.0. In addition, the diameter of the regenerate tail is less than the diameter of the original tail and pigmentation has yet to occur. As a result, the tail remains pink in colour (Figures 8A and 8B). Although the external gross morphology is basically uniform throughout stage V, serial histology reveals two distinct substages of structural organization.

#### Histology - Early Stage V

In early stage V the wound epithelium remains comparatively thick (7-12 layers of cells), stratified and lies in direct contact with the underlying blastema (Figure 8C and 8D). There is no dermis present at this stage. Although the regenerate tissue consists largely of blastema cells invaded by a network of capillaries (Figure 8C), various cells are beginning to undergo differentiation (see below).

Outgrowth of the ependymal tube matches continued growth of the regenerate tail (Figure 8C), with the ependymal ampulla remaining a near constant distance from the overlying wound epithelium. Axons passing from dorsal root ganglia of the original tail continue to invade the blastema. These axons parallel the ependymal tube. In addition to capillaries, various larger diameter vessels appear in early stage V.

Skeletal regeneration becomes increasingly obvious beginning at early stage V as a cone-like condensation of cells forms around the ependymal tube and begins depositing extracellular matrix (Figures 8C and 8E). This matrix stains positive for Alcian blue (Figure 8E), indicating the presence of glycosaminoglycans, characteristic of cartilage. Throughout the remainder of regeneration this cone demonstrates a conspicuous proximal to distal axis of maturation, with the most differentiated tissues occurring immediately adjacent to the original tail. Concurrent with skeletogenesis, segments of musculature continue to differentiate. Proximally myoblasts are beginning to fuse, forming myotubes, and have become organized into myomeric segments separated by zones rich in connective tissue (presumptive myosepta). In transverse section the arrangement of presumptive skeletal muscles lack the eight-fold organization of the original and are no longer divided by vertical and horizontal septa into quadrants. Instead, regenerating muscles are organized into 14-16 small bundles arranged in a circle around the cone-like condensation and ependymal tube (Figure 8F).

PCNA positive cells occur throughout the regenerating tail including the basal layers of the wound epithelium, the cells of the blastema, the cells of the cone-like skeletal condensation surrounding the ependymal tube and cells of the presumptive musculature. In addition, cells in the ependymal tube, endothelial cells and cells associated with axons also stain positively for PCNA.

#### Histology - Late Stage V

In late stage V the outer layer of the wound epithelium becomes keratinized and epidermal scales begin to form. Scalation begins as the basal layer of the wound epithelium invades the underlying blastema, creating a regular series of ingrowths or ridges (Figure 8G). These ridges are obliquely oriented, with the deepest margins directed away from the caudal tip of the tail. At this stage the dermis remains undefined. Within the blastema an increasing number of tissues are differentiating, especially towards the proximal contact with the original tail.

Among the new blood vessels is a large diameter artery continuous with the caudal artery of the original tail. Unlike the original caudal artery, this new vessel lacks arterial sphincters and the thickness of the walls is comparatively thinner than those of the original vessel.

One of the most obvious differences between early and late stage V is the state of skeletal development. Cells of the cone-like condensation have begun to differentiate into chondroblasts and chondrocytes and the investing extracellular matrix stains intensely positive for Alcian blue (Figure 8H). Furthermore, late stage V marks the first appearance of Sox9 expression in both the original and the regenerating tails. Sox9 is a transcription factor required for chondrocyte differentiation [Crombrugghe et al., 2000] and hence cartilage formation. Sox9 positive cells are found throughout the differentiating skeletal cone (Figure 8I). In addition, Sox9 expression is also observed in chondroblasts/-cytes of the original tail, ependymal cells of the regenerating and original tail, and some of the cells of the blastema. Throughout late stage V the regenerate tail still demonstrates a proximal to distal axis of maturity, with tissue closest to the original tail having differentiated and more distal populations still appearing as blastema cells. In parallel with maturation of the skeleton, muscle differentiation continues in a proximal to distal sequence. Compared with the original skeletal muscle, regenerating myotubes are relatively small in diameter and have centrally positioned nuclei.

In the regenerating portion of the late stage V tail PCNA positive cells are observed in the majority of the tissues, including throughout the regenerating ependymal tube, developing cartilaginous cone and axial musculature. Basal cells within the regenerate epithelium also continue to proliferate.

### Stage VI (18 - 30 days post-autotomy)

#### Overview

**Figure 9:**
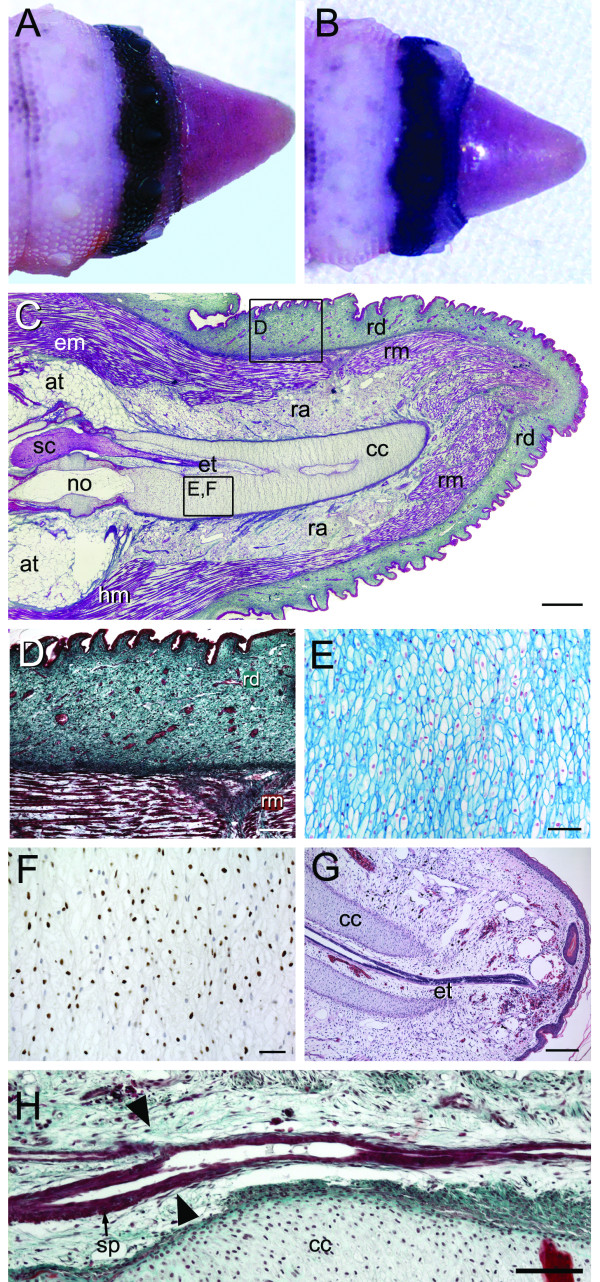
**Stage VI of tail regeneration**. Stage VI of tail regeneration. *Eublepharis macularius*. **(A,B) **Gross morphology of regenerating tail in lateral **(A) **and dorsal view **(B)**. The regenerating tail is tapering cone with a diameter: length approaching 1.0. Serial sections (dorsal toward the top) stained with Masson's trichrome **(C,D,H)**, Alcian blue **(E)**, immunostained with Sox9 **(F) **or stained with hematoxylin and eosin **(G)**. **(C) **Sagittal section of tail. Note that most tissues have fully differentiated and an aggregation of blastema cells is no longer apparent. The cartilaginous cone is anchored to the remnant of original vertebra. **(D) **Closer view of the region identified in panel **(C) **taken from a different section. By this stage of regeneration the integument of includes the newly formed dermis. **(E,F) **Closer view of the region identified in panel **(C) **taken from a different section. The cartilaginous cone is cell-rich and stains strongly positive for Alcian blue. Differentiating chondrocytes are immunostained (brown) for Sox9. **(G) **Sagittal section through the distal tip of the tail demonstrating the ependymal tube (stained red) passing through the cartilage cone (stained pink). **(H) **Longitudinal section through the caudal artery (stained red) of the original tail and regenerate tail. **Arrowheads **indicate the transition from the original (on the left) to the regenerate caudal artery (on the right). ca, caudal artery; cc, cartilage cone; em, epaxial muscle; et, ependymal tube; hm, hypaxial muscle; no, notochord; ra, regenerated adipose tissue; rd, dermis; sc, spinal cord; sp, arterial sphincter. Scale bars: c = 500 μm; d,e = 20 μm; f,g = 100 μm; h = 50 μm.

In stage VI the regenerating tail forms a tapering cone with a diameter to length ratio of approximately 1.0 and a conspicuous pattern of uniform scalation. However, the diameter of the regenerate tail is still less than that of the original tail and the regenerate lacks pigmentation (Figures 9A and 9B).

#### Histology

In section the wound epithelium is now the same thickness as the epidermis of the original tail (approximately 4-7 cell layers thick) and overlies a newly differentiated dermis (Figures 9C and 9D). Hereafter, the wound epithelium does not appear distinct from the epidermis of the original tail.

By stage VI most of the regenerating tissues have differentiated and the uniform mass of cells that constitute the blastema is no longer present (Figure 9C). The regenerate skeleton consists of a well-defined cone that stains intensely with Alcian blue (indicating the presence of glycosaminoglycans) and is positive for Sox9 (Figures 9E and 9F). There is no evidence of a regenerated notochord. The ependymal tube is localized within the hollow centre of the cartilaginous cone, and is typically displaced slightly dorsal to centre. In transverse section the ependymal tube consists of a monolayer of ependymal cells encircling a hollow central canal (Additional file 5). The terminal end of the ependymal tube, the ependymal ampulla, is located immediately distal to the tip of the cartilaginous cone (Figure 9G). Coursing with the ependymal tube within the center of the cartilage cone are various blood vessels. The cartilaginous cone is also surrounded by a number of peripheral axons and blood vessels. These axons can be traced back to dorsal root ganglia of the original tail. The regenerate continuation of the caudal artery passes ventral to and parallel with the cartilage cone. Unlike the original caudal artery, the regenerate vessel lacks sphincters (Figure 9H).

Tissue differentiation is nearly complete at stage VI and it is clear that the regenerated adipose tissue and axial musculature are not divided into quadrants. However, the relationship between the tissues remains identical with adipose-rich tissue encircling the skeleton, surrounded in turn by regenerated musculature. Unlike the original tail, there are no obvious tendons attaching the musculature to the skeleton, although connective tissue myosepta are present subdividing the axial musculature.

Numerous PCNA positive cells are present in the basal layers of the epidermis and within the regenerating dermis, as well as within the regenerating muscle, adipose tissue, ependymal tube and cartilaginous cone, although in the latter tissue cell proliferation appears to have diminished compared to earlier stages. Sox9 positive cells are primarily associated with the cartilaginous cone with additional expression by cells of the ependymal tube and fibroblast/mesenchyme-like cells within the adipose tissue and dermis.

### Stage VII (25+ days post-autotomy)

#### Overview

**Figure 10:**
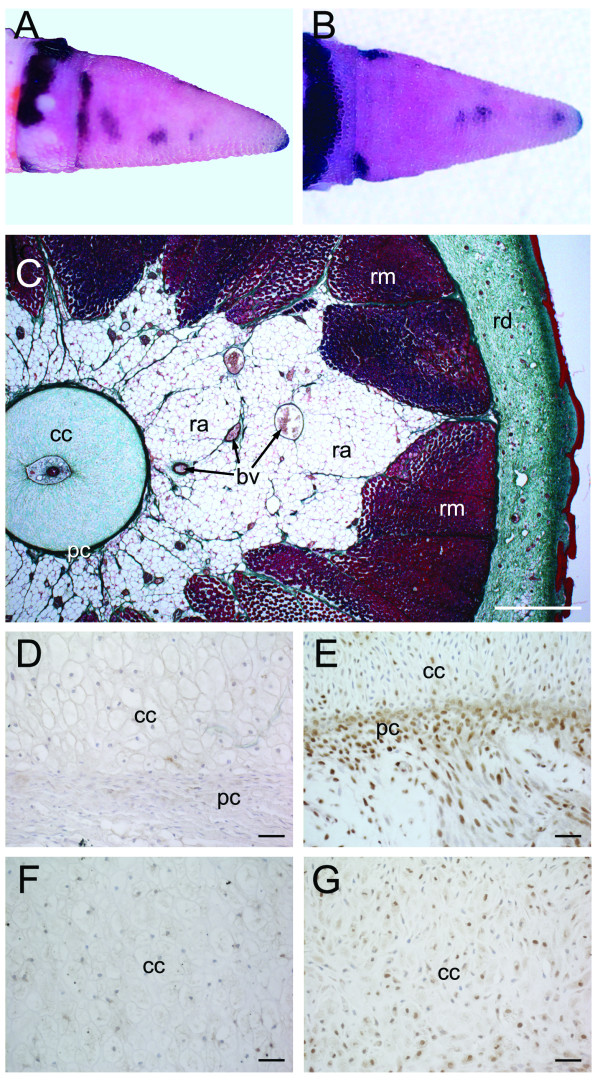
**Stage VII of tail regeneration**. Stage VII of tail regeneration. *Eublepharis macularius*. **(A,B) **Gross morphology of the regenerate tail in lateral **(A) **and dorsal view **(B)**. The regenerate tail is tapering cone that is longer than wide (length: diameter greater than 1.0). Serial sections stained (dorsal toward the top) with Masson's trichrome **(C)**, immunostained with PCNA **(D,E) **or immunostained with Sox9 **(F,G)**. **(C) **Transverse section through the regenerate tail. Similar to the original tail (see figure 2C), the regenerate appendage is concentrically organized with a central nervous system (ependymal tube, stained pink) enclosed by a regenerated skeleton (cartilaginous cone, stained green), surrounded by regenerated adipose tissue (unstained), regenerated skeletal muscle (stained red) and regenerated integument (dermis, stained green; epidermis, stained red). **(E,F) **Closer view of the interface between the cartilaginous cone and surrounding perichondrium immunostained for PCNA (brown), indicating proliferating cells, and counterstained with hematoxylin (blue). As the tissues differentiate and mature cell proliferation is reduced. In proximal portions of the tail **(D)**, there are fewer PCNA-positive cells compared to more distal positions **(E)**. **(F,G) **Closer view of the cartilaginous cone immunostained for Sox9 (brown), identifying differentiating chondrocytes, and counterstained with hematoxylin (blue). Chondrocytes in the proximal portion of cartilaginous cone **(F) **are fully differentiated and no longer express Sox9. Chondrocytes in the distal portion of cartilaginous cone **(G) **are positive for Sox9, indicating they are still undergoing the early phases of differentiation. cc, cartilage cone; pc, perichondrium; ra, regenerated adipose tissue; rd, regenerated dermis; rm, regenerate muscle. Scale bars: c = 500 μm; d = 200 μm; e-h = 20 μm.

Three morphological features characterize stage VII: the tail has acquired a tapering cone shape with a length to diameter ratio greater than 1.0; the width of the proximal portion of the regenerate tail is equivalent to that of the adjacent original tail; and the regenerate tail has developed pigmentation. Early during stage VII pigmentation is restricted to a number of grey spots against an otherwise pink regenerate tail. These spots appear primarily across the dorsal and lateral surfaces of the tail (Figures 10A and 10B). Later in stage VII the spots blacken, and the background colour is replaced by white and grey similar to the rest of the body. By stage VII virtually all the tissues have differentiated and the tail has lengthened and closely resembles its original form.

#### Histology

The integument of the regenerate tail is well organized into a covering of uniform scales and consists of both a stratified epidermis and a connective-tissue rich dermis. Whereas the regenerate epidermis is the same thickness as that of the original, the regenerate dermis is slightly thicker. The interface between the regenerate and original tails is well defined by the continued presence of epidermal downgrowths.

The regenerate skeleton consists of a chondrocyte-rich, matrix-poor cartilaginous cone (Figure 10C) that attaches directly to the quarter vertebral remnant. The distal tip of this structure remained open even in an individual allowed to regenerate for 70 days. It remains unclear if the distal tip of the cartilage cone will eventually close as has been reported-for other species [3]. The cartilaginous cone is surrounded by perichondrium along both its external and internal surfaces. The perichondrium of the regenerate skeleton is continuous with the periosteum of the remnant caudal vertebra.

The ependymal ampulla is located just deep to the dermis at the distal tip of the tail and many peripheral axons are present within the adipose tissue. The once pervasive capillary network has matured into an organized architecture of various large multilayered vessels. A continuation of the caudal artery passes the length of the regenerate tail, ventral to the cartilage cone.

By stage VII, PCNA expression in the regenerate tail was observed primarily in the basal layer of the epithelium and some cells of the dermis. Proliferation in all other tissues demonstrates a proximal to distal gradient, with relatively few PCNA positive cells proximally and larger numbers of proliferating cells distally (Figures 10D and 10E). Sox9 expression continues in stage VII, although the number of positive cells has diminished. Sox9 is largely restricted to cells of the cartilage cone and a limited number of cells in the ependymal tube, adipose tissue and dermis. Similar to PCNA expression, Sox9 demonstrates a higher level of expression distally than proximally (Figures 10F and 10G).

## Discussion

Tail regeneration in *E. macularius *follows a conserved sequence of morphological and histological events comparable with observations made on other lizards (e.g., [3,8-12]). Based on recurrent features of external gross morphology, tail regeneration is divisible into seven distinct stages. Stages I - III focus on wound healing while IV - VII document changes in blastema size and shape, and growth of the new tail. Each morphological stage also matches with major histological events including clot formation, wound repair, blastema proliferation and tissue morphogenesis. Using recurrent histological features, two substages of stage V (early and late) are recognized. A summary of the stage of initial onset and duration of major events during tail regeneration is depicted in Table 2. Ultimately, the fully regenerated tail (stage VII) is superficially similar to the original in general appearance, including pigmentation, but differs in details of scalation, tissue organization and structure of the axial skeleton and central nervous system (Additional file 6). Notably, a cartilaginous cone replaces the original skeleton and an ependymal tube replaces the original spinal cord.

**Table 2:** Summary of the onset and duration of major events during tail regeneration in *Eublepharis macularius *.

	I	II	III	IV	V	VI	VII
	(3 hrs - 1 d)	(18 hrs - 8 d)	(4 - 8 d)	(8 - 15 d)	(12 - 26 d)	(18 - 30 d)	(25+ d)
cell proliferation							
clot							
blastema							
re-epithelialization							
neo-vascularization							
nervous tissue							
regeneration							
muscle regeneration							
cartilage regeneration							
Sox9 immunostaining							
	

### Wound Healing

At the site of voluntary tail loss lizards undergo wound healing without the formation of scar tissue. Wound healing begins immediately following autotomy and concludes with the formation of a complete wound epithelium. In stage I the autotomy surface resembles a large cross-sectional wound with a prominently exposed autotomized vertebra. Blood loss from the major arterial vessel, the caudal artery, is minimal as a result of rapid closure of the arterial sphincter proximal to the autotomy surface. A small amount of blood leaking from the spinal artery gives rise to a blood clot capping the ruptured end of the spinal cord. Concurrent with haemostasis, the autotomy surface is secondarily reduced by contraction of the adjacent epidermis.

By stage II the once localized clot has expanded across the entire autotomy surface. This clot is a temporary structure that is composed of blood cells, tissue fluid and tissue debris, but no newly formed collagen fibers. Hence, wound healing at the autotomy surface does not involve the formation of a fibrotic scar. Deep to the clot, cell proliferation begins within both the epidermis surrounding the autotomy surface and a distinct population of cells distal to the severed spinal cord. This mass represents the earliest formation of the blastema. In urodeles and zebrafish, blastema formation is not observed until after closure of the wound site by the wound epithelium [21,22]. This difference may, however, be related to the more rapid wound closure seen in these organisms.

Simultaneously with development of the wound epithelium, multinucleated osteoclasts and chondroclasts begin resorption of bone and cartilage associated with the protruding vertebra. Wound healing is achieved by stage III with the complete reepithelialization of the autotomy surface and ablation of the exudate clot. The newly formed wound epithelium continues to proliferate and thicken (up to 12 cell layers thick compared to 4-7 in the original epidermis), especially at the apical epithelial cap (AEC). In other regeneration models (e.g. urodeles, teleosts) the AEC has a well-documented role as a source of morphogenetic information comparable with the apical epithelial ridge of limb development [23,24]. For example, during axolotl limb regeneration the wound epithelium is the primary source of the patterning protein Msx2 [25] and the extracellular matrix remodeling protease MMP9 [26]. During zebrafish tail fin regeneration the patterning molecule Sonic hedgehog (Shh) is expressed exclusively by basal epithelial cells that overlie the blastema [27,28]. Although details of the morphogenetic role of the AEC in lizards remains unclear, previous experimental studies have demonstrated that destroying the AEC [12], replacing the AEC with a graft of mature skin [29] or removal of the AEC [30] blocks regeneration.

In *E. macularius *the period of wound healing varies, taking 3-8 days, but is similar overall to the timeframe reported for other lizards (e.g., 7-9 days for *Anolis carolinensis *[11]; 5-7 days for *Podarcis muralis *[31]). Interestingly, comparable rates of wound healing (to the point of reepithelialization) have been documented following amputation of the digit tip in laboratory mice and Rhesus monkeys (*Mus musculus *6-8 days [32]; *Macaca mulatta *7-10 days [33]). In contrast, wound closure following amputation of the limb or tail in regenerating urodeles is very rapid, only 4-24 hours [34]. The location of the wound epithelium also differs between urodeles and lizards. In lizards the wound epithelium forms deep to the clot and damaged tissue are ablated [3,8], whereas in urodeles the wound epithelium encloses the clot, and damaged tissues are degraded internally by macrophages and neutrophils [34].

Unlike wound healing that leads to regeneration, healing by fibrosis (scar formation) is characterized by a strong inflammatory response and the rapid production of an acellular fibrous matrix - the mature scar [35]. Whereas these events classically define scar formation during mammalian wound healing, they have also been observed following amputation of the limb in lizards [12,36]. More specifically, the wound healing response to limb amputation includes a prolonged period of leukocyte infiltration into tissues adjacent to the wound site (primarily heterophils, eosinophils and macrophages), the rapid formation of a basement member and the retention of granuloma tissue [36]. Combined, these events are interpreted to promote fibrosis, limit epithelial-mesenchymal interactions and inhibit blastema formation [36].

Scar-free wound healing is relatively rare in mammals, although several examples have been documented (e.g., digit tip regeneration in rodents and primates, ear hole replacement in lagomorphs, regeneration during embryonic development) [37-39]. As for the autotomized gecko tail, mammalian scar-free wound healing undergoes re-epithelialization to form a wound epithelium. However, unlike geckos this wound epithelium does not thicken to form an AEC. To date there is no evidence that mammalian wound epithelium acts as a source of morphogenetic information [33].

Given the obvious parallels between mammals and lizards in wound repair response leading to scar formation, we are in strong agreement with previous suggestions [12] to further explore wound healing in lizards as a biomedical model to better understand scar-free repair.

### Blastema Formation

Blastema formation begins with the aggregation of a uniform population of blastema cells located distal to the retracted spinal cord and deep to the exudate clot (Stage II). PCNA immunohistochemistry reveals this cell population to be highly proliferative. Significantly, in *E. macularius *the blastema first appears prior to the completion of the wound epithelium. Typically, blastema formation in urodeles does not begin until after the wound is closed. However, it has been noted that in urodeles such as *N. viridescens*, initial reentry of cells into the cell cycle is not dependent on the presence of the wound epithelium [40].

Cell proliferation continues throughout stages III-V, with an increase in blastema size matched by the development of new blood vessels and the outgrowth of the ependymal tube (see below). Beginning at stage V the once uniform population of cells in the blastema begins to differentiate, resulting in the formation of cartilage, muscle, adipose tissue and fibrous connective tissue. Differentiation proceeds in a cranial to caudal axis, with the most mature tissues in the proximal region of the regenerate tail. Although cell proliferation continues during this time the cell population is no longer uniform and the blastema ceases to be discretely recognizable. Congruent with the differentiation of cell types, levels of proliferation are highest distally and lowest proximally.

At first glance our results appear to contradict previous work using autoradiography, which suggested that cell proliferation was initially limited in the blastema (e.g., [11]). However we caution against making direct comparisons at this time, as our investigation differed from these earlier studies in terms of the model species employed (e.g., [11] investigated *Anolis carolinensis*, a lizard that is phylogenetically quite distant from geckos) and details of the husbandry conditions (e.g., some *A. carolinensis *were housed in groups, others were not). It is also worth noting that direct intraspecies comparisons of cell proliferation during regeneration using PCNA immunostaining and bromodeoxyuridine (BrdU) labelling have yielded similar results [17].

Whereas these results provide important information about the pattern of blastema formation, growth and differentiation, the source of the blastema and the interaction between this cell population and the overlying wound epithelium (and AEC) remains unclear. Most previous studies have suggested that cells of the blastema arise from dedifferentiation of tissues within the original tail stump and/or from a population of quiescent stem-like cells [10,31,40]. Recently, Kragl and colleagues traced cell fates during regeneration using juvenile green fluorescent protein axolotls [41]. Significantly, they determined that most cells within the blastema have a restricted fate (i.e., they are not multipotent) and do not switch between cell types originating from different embryonic germ layers during regeneration [41]. They also noted that some cells (e.g. cartilage derived cells) retain a memory of their original positional information and thus occupy specific locations within the blastema and regenerate tail [41]. Although these findings are presently limited to juveniles of a single urodele species (*Ambystoma mexicanum*), they provide strong support that blastema cells have a restricted lineage.

### Vasculature and Nervous Tissue

Blood vessels and nervous tissue (i.e., the ependymal tube and peripheral axons) invade the blastema shortly after closure of the wound epithelium (Stage III). Similar to urodeles and teleosts, these structures are not formed de novo but result from the outgrowth of original tissues within the proximal (non-autotomized) portion of the tail [41,42]. Unlike urodeles, in geckos the early forming blastema is rich in blood vessels; in urodeles the early forming blastema is relatively avascular [42,43]. However, in other regenerative model systems such as the zebrafish, angiogenesis has been noted to occur soon after amputation [44]. By the later stages of gecko tail regeneration (stages IV-VI) larger multilayered vessels develop, including a continuation of the caudal artery. Regenerated blood vessels do not develop sphincters suggesting that the new tail is poorly adapted for subsequent amputations.

Related to its demonstrated importance in other blastema-mediated regeneration models, rapid outgrowth of the nervous system is critical to blastema formation [1,12,29,45]. In *E. macularius*, as for other lizards, the most obvious components of the regenerating nervous system are the ependymal tube and axons derived from the proximal portion of the original spinal cord and dorsal root ganglia. Dorsal and ventral gray columns and dorsal root ganglia are not regenerated. Although difficult to discretely identify at the level of light microscopy, descending tracts from the original spinal cord are reported to closely bundle around the ependymal tube [3,10,12,46,47]. The ependymal tube develops as a caudal extension of epithelial cells surrounding the central canal of the spinal cord. As demonstrated by PCNA analysis, ependymal cells actively proliferate as the ependymal tube extends towards the AEC. Ultimately, the regenerated skeletal system (a cartilaginous cone) encloses the ependymal tube. A similar pattern of ependymal tube regeneration is observed in urodeles [19,48]. However unlike lizards, regeneration of ependymal tube in urodeles is followed by the complete restoration of the spinal cord. Accordingly, it is hypothesized that ependymal tube development in geckos represents a truncated version of the process demonstrated by urodeles.

In parallel with ependymal tube outgrowth, axons begin to regenerate at stage III. These axons pass caudally to enter the blastema and gradually become associated with differentiating muscles, connective tissues and skin. Axonogenesis matches outgrowth of the blastema - as the regenerate tail length increases so does the length of the axons. Consequently, regenerate tissues receive sensory and motor innervations from the original tail [3,10,12,46,47,49].

It is well established that regeneration is nerve-dependent [3,10,12,46,47,49-52]. For tail regeneration, the central nervous system appears to play a crucial role. For example, removal of the spinal cord proximal to the site of tail loss inhibits regeneration whereas removal of the proximal-most dorsal root ganglia does not [29,51]. Furthermore, regeneration can be rescued by ependymal tube grafts [29,51]. Based on these observations it is widely accepted that the central nervous system secretes one (or more) trophic factor(s) necessary to induce tail regeneration. In the newt it has been demonstrated that the protein nAG will rescue regeneration in denervated limbs [52]. Whereas the identity of a comparable trophic factor in lizards remains unknown, a number of candidate molecules have been proposed (e.g., basic fibroblast growth factor [53]).

### Muscle and Skeletal Re-development

Regeneration of striated muscle and the axial skeleton does not begin until the blastema has begun to bulge prominently from the original tail stump (late Stage IV- Stage V). Muscle formation is first observed as aggregations of mononucleated mesenchymal-like myoblast cells in the proximal-most regions of the regenerate tail. Myoblasts within these condensations alter their morphology, becoming elongate and spindle-shaped, and then align with one another. Fusion of aligned myoblasts gives rise to myotubes and is matched by the development of connective tissue-rich myosepta. Unlike the epaxial and hypaxial organization of the original tail, early regenerating muscles consist of 14-16 symmetrically arranged bundles surrounding the skeletal condensation (Figure 8F). As regeneration continues, muscle bundles begin to develop in more caudal positions within the regenerate tail and the once immature myotubes differentiate into segmented muscle fibres. A similar proximal to distal pattern of muscle differentiation has also been observed in urodele tail and limb regeneration [15,48].

The regenerated skeleton begins as a cone-like condensation of chondroprogenitor cells developing around the outgrowing ependymal tube. Similar to muscle (and adipose tissue) regeneration, maturation of the cartilaginous cone occurs in a proximal to distal manner. Overall, regenerate tail cartilage is highly cellular although the extracellular matrix present is rich in glycosaminoglycans as evidenced by positive Alcian blue histochemistry. Once completely regenerated, the outermost and innermost layers of the cartilaginous cone may calcify but bone is never developed [3]. At least initially, the process of skeletal regeneration in lizards resembles that of urodeles with the formation of a cartilaginous cone from a condensation of blastema cells. Unlike lizards, urodeles gradually segment and ossify the cone, giving rise to bony vertebra [45]. This observation suggests that skeletal regeneration in geckos may represent a truncated version of the pathways employed by salamanders and newts.

Our study determined that regenerate cartilage is identifiable histochemically (with Alcian blue and Safranin O; early Stage V) prior to Sox9 expression (late Stage V). During embryonic development in mammals Sox9 has a well-documented role in chondrogenesis before (and after) condensation formation (e.g., [19,20]), and thus identifies presumptive cartilage in advance of extracellular matrix secretion. The unexpected delay in Sox9 expression (i.e., post-extracellular matrix secretion) may reflect an evolutionary difference in chondrogenesis between lizards and mammals. Alternatively, this delay may be related to the hypothesis that blastema cells are cell-type-specific [54]. Hence, if regenerated cartilage comes from a cell population with a restricted cartilage fate then the initial role of Sox9 as determinant of chondroprogenitors may be redundant.

Sox9 expression by regenerate cartilage cells continues throughout stages VI and VII. By stage VII Sox9 expression had diminished in a characteristic proximal to distal gradient. Sox9 expression in mature regenerate chondrocytes is minimal. Interestingly, Sox9 expression was also observed in chondrocytes of the original tail and various other cell types in the regenerate tail including ependymal cells and blastema cells but only beginning at late stage V; prior to this no cells, original or regenerate, express Sox9. In addition to its role in chondrogenesis, Sox9 has been identified as marker of migrating cells such as neural crest cells [55]. However, it remains unclear why Sox9 is not observed in these populations until late stage V, especially since cell migration is predicted to play a role in blastema formation. In mammals, Sox9 is also expressed in neuronal precursors including pre-oligodendrocytes [55].

Geckos, similar to larval urodeles [56], do not regenerate the notochord [3,8,29]. In contrast, the notochord does regenerate in anuran tadpoles. In tadpoles, cells from the amputated notochord proliferate and replace the missing tissue [57]. Although not the focus of the current study, it is worth noting that we did not find evidence that notochordal cells proliferate or otherwise participate in tail regeneration.

## Conclusions

This study of tail regeneration in lizards provides a critical contribution to our understanding of epimorphic regeneration in amniotes. To date, most investigations of naturally occurring blastema-mediated regeneration focus on non-amniote model systems, in particular teleost fins and urodele limbs. Although these taxa demonstrate perfect regeneration, they are distantly related to mammals and hence their application to biomedical studies remains uncertain. Our investigation targeted a representative lizard to bridge the evolutionary gap between poorly regenerating mammals and perfectly regenerating nonamniote models. We show that the process of tail regeneration in lizards is a highly organized phenomenon that results in the complete, albeit non-identical, restoration of a complex multitissue structure. Many of the events observed during epimorphic tail regeneration in lizards are conserved with those of urodeles and teleosts. Furthermore, tail regeneration also shares various characteristics with rare instances of mammalian reparative regeneration (e.g., rodent digit tip regeneration). For example, all these events require the development of a wound epithelium and blastema, and all take place in the absence of scar formation. We conclude that the major events of epimorphic regeneration are highly conserved across vertebrates and that a comparative approach is an invaluable tool for regenerative medicine.

## Methods

### Animal Housing and Use

All *E. macularius *were captive bred and obtained from one of two commercial suppliers (Pat Wise Lizards, Calgary, Alberta, Canada; Global Exotic Pets, Kitchener, Ontario, Canada). At the start of the experiment, all were less than one year in age with an average total body length (snout to vent plus tail) of 123.5 mm (range 88-172 mm; Additional file 1). Animal care protocols were approved by the University of Guelph Animal Care Committee (Protocol Number: 09R026). In total 89 *E. macularius *were used in three separate experimental trials. Animal husbandry follows the work of Vickaryous and McLean [6] (see also [5]). Briefly, geckos were housed individually in standard rat-sized polycarbonate enclosures in an isolated room with a 12:12 photoperiod, an ambient humidity of 40-50% and a room temperature of ~24°C. In order to facilitate behavioural thermoregulation, a temperature gradient was created using a subsurface heating cable (Zoo Med's Repti Heat Cable) under one end of the enclosure set to 28°C. Animals were fed daily a diet of gut-loaded mealworms (larval *Tenebrio *spp.) dusted with powdered calcium and vitamin D3 (cholecalciferol) supplement.

### Tail Collection

Tail collection involved inducing autotomy. Individuals were randomly assigned into control (no autotomy) and experimental (autotomy) groups. On experimental day one, autotomy was induced in all experimental individuals by firmly grasping the tail between the thumb and index finger and applying pressure. In the first instance a position halfway between the cloaca (vent) and the tail tip was selected and grasped as described. The gecko autotomizes the distal portion of the tail (at the level of the finger-applied pressure) but retains the more proximal half. Following this initial autotomy event individuals were allowed to regenerate. Tails were then induced to autotomize a second time to provide tissue samples at each of the seven main morphological stages (I-VII). For the second autotomy event, the original tail was grasped (as described) at a more proximal position representing roughly the majority of the tail. This second event resulted in the collection of regenerating tissue plus a segment of original tail.

### Histochemistry

Immediately following autotomy, tails (original and original/regenerate) were fixed in either 10% neutral buffered formalin (NBF; Protocol Supplies) or 4% paraformaldehyde (PFA; paraformaldehyde in 1× phosphate buffered saline [PBS]) for 24 hours. Following fixation, tails were rinsed in distilled water and stored in 70% ethanol prior to processing. Preparation for serial histology included decalcification in CalEx (Fisher Scientific) for 30 minutes and tissue processing (dehydration to 100% ethanol, clearing in xylene, embedding in paraffin wax; Ventana Renaissance Tissue Processor). Serial sections were cut at 5 μm on a rotary microtome and mounted on charged slides (Snow Coat X-tra, Surgipath). Following the staining protocols (see below), sections were coverslipped with cytoseal (Fisher Scientific).

Various histochemical stains were used to identify the tissue and cellular compositions of the tails, including hematoxylin and eosin [58], Safranin O [59], Alcian blue [60] and a modified Masson's trichrome. Hematoxylin and eosin (H&E) is a routine two-colour histochemical technique that stains nuclei blue (with hematoxylin) and cytoplasm, collagen and red blood cells pink (with eosin). Safranin O and Alcian blue are cationic dyes used to visualize glycosaminoglycans (red with Safranin O, blue with Alcian blue), a characteristic component of the extracellular matrix of cartilage. Safranin O is counterstained with Fast Green, a bluish-green connective tissue dye. Masson's trichrome is a multi-colour connective tissue stain that visualizes collagen fibres and cartilage as green, muscle and epidermis as red, bone as red with green mottles, and the spinal cord as pinkish-red. The protocol for Masson's trichome was adapted from Witten and Hall [61]. Briefly, sections were deparaffinized through a series of three xylenes (2 minutes each) and then rehydrated through three changes of absolute isopropanol (2 minutes each), 70% isopropanol (2 minutes) and deionized water (dH_2_O; 2 minutes). Sections were then stained in Mayer's hematoxylin (10 minutes), rinsed in running water, blued in ammonia water and stained with ponceau xylidine/acid fuchsin (2 minutes). After rinsing, sections were placed in phosphomolybdic acid (10 minutes), rinsed, stained in 0.02% light green (90 seconds) and rinsed again. Slides were then dehydrated through 95% isopropanol (2 minutes), three changes of absolute isopropanol (2 minutes each) and three changes of xylene (2 minutes each), prior to coverslipping.

### Immunohistochemistry

Immunohistochemistry was carried out as described in Vickaryous and McLean [6]. Briefly, tissues were fixed with either 10% NBF or 4% PFA and prepared for serial sectioning as described above. Slide mounted serial sections were deparaffinized, rehydrated and then quenched with 3% hydrogen peroxide in dH_2_0 for 10 minutes to eliminate endogenous peroxidases. Slides were then rinsed three times with PBS and blocked with 3% normal goat serum in PBS (Jackson ImmunoResearch Laboratories, Inc.) for 1 hour at room temperature to prevent non-specific binding. Blocking solution was tipped off and slides were incubated overnight at 4°C in a humidified chamber with primary antibody. Primary antibodies utilized were rabbit polyclonal anti-PCNA (1:500 in PBS, Santa Cruz Biotechnology, Inc.) and rabbit polyclonal anti-Sox 9 (1:600 in PBS, Abcam). Control slides substituted sterile PBS for primary antibody. Slides were then rinsed three times with PBS and incubated with secondary antibody (Goat anti-rabbit IgG, 1:500 in PBS, Jackson ImmunoResearch Laboratories, Inc.) in a humidified chamber for 1 hour at room temperature. Slides were again washed three times with PBS and then incubated with peroxidase-conjugated streptavidin (1:200 in PBS, Jackson ImmunoResearch Laboratories, Inc.) in a humidified chamber for 1 hr at room temperature. Following incubation, slides were washed three times with PBS, stained with 3,3'-diaminobenzidine (DAB: 200 ml dH_2_0, 2 mL DAB, 300 μl hydrogen peroxide) for 1 minute and rinsed in running water. Mayer's hematoxylin (5 dips) was used to counter stain slides, followed by bluing in ammnonia water, as for H&E staining. Following dehydration and clearing with xylene, sections were coverslipped with cytoseal.

## Authors' contributions

KEM performed the histology, histochemistry and immunohistochemistry, participated in experimental design, data collection and analysis, and drafted the manuscript. MKV conceived the study, participated in experimental design, data collection and analysis, and writing the manuscript. All authors read and approved the final manuscript.

## Supplementary Material

Additional file 1 **Supplementary Table 1: Initial body size (snout-vent, tail, and total body length) and tail as a percentage of length for *Eublepharis macularius *used in this study**. A list of the starting body size measurements for all 89 *E. macularius *taking part in this experiment. The experiment was conducted as three separate trials indicated by the specimen numbers EM 9 5 # (May 2009), EM 9 6 # (June 2009) and EM 10 2 # (February 2010). Click here for file

Additional file 2 **Supplementary Figure 1: Original anatomy of the gecko tail in sagittal section**. ***Eublepharis macularius***. Serial section stained with Masson's trichrome (dorsal towards the top of the page; distal towards the left of the page). The spinal cord (stained pink) is centrally positioned. Ventral and parallel to the spinal cord is the notochord. Ventral to the vertebral column is the caudal artery (stained red). Vertebrae are surrounded by bands of adipose tissue (unstained) and skeletal muscle (stained red). The integument is composed of a thick layer of dermis (stained green) encircled by the epidermis (stained red). at, adipose tissue; ca, caudal artery; ch, chevron; cv, caudal vein; de, dermis; em, epaxial musculature; hm, hypaxial musculature; id, intervertebral disc; nc, notochordal cartilage; no, notochord; ns, neural spine; sc, spinal cord. Scale bar = 500 μm. Click here for file

Additional file 3 **Supplementary Figure 2: Myomere organization of the original tail**. ***Eublepharis macularius***. Gross specimen visualized with two drops of commercial red food colouring (Allura red and Erythrosine B) (dorsal towards the top of the page; distal towards the left of the page). Skeletal muscles in the tail are arranged into a prominent series of W-shaped zigzag interdigitations (white lines). Scale bar = 2 mm. Click here for file

Additional file 4 **Supplementary Figure 3: Cell proliferation in the original tail at stages II, III and IV**. ***Eublepharis macularius***. **(A-D) **Longitudinal sections immuostained with PCNA (brown; indicated with black arrows) and counterstained with hematoxylin (blue). **(A,B) **Stage II. **(A) **Basal germinative cells of the epidermis. **(B) **Ependymal cells of the ependymal tube. **(C,D) **Stage III. **(C) **Basal germinative cells of the epidermis. **(D) **Ependymal cells of the ependymal tube. **(E,F) **Stage IV. **(E) **Basal germinative cells of the epidermis. **(F) **Ependymal cells of the ependymal tube. bg, basal germinative cell; de, dermis; et, ependymal tube. Scale bar = 20 μm. Click here for file

Additional file 5 **Supplementary Figure 4: Regenerated ependymal tube**. ***Eublepharis macularius***. Transverse serial section stained with Masson's trichrome (dorsal towards the top of the page). The ependymal tube (stained pink) is centrally positioned, enclosed within the regenerated cartilaginous cone (stained green). Also within the cartilaginous cone are various blood vessels. bv, blood vessel; cc, cartilaginous cone; et, ependymal tube. Click here for file

Additional file 6 **Supplementary Figure 5: Comparison of original and fully regenerated gecko tails in transverse section**. ***Eublepharis macularius***. Serial sections stained with Masson's trichrome (dorsal towards the top of the page). **(A) **Original tail. The tail is concentrically organized, with the spinal cord (stained pink) enclosed within the neural canal of a vertebra (in this view stained red with green mottles). Ventral to the spinal cord is the notochord. The vertebra is enclosed by longitudinal bands of perivertebral adipose tissue, and then skeletal muscle (stained red). **(B) **Stage VII regenerated tail. Similar to the original tail, the regenerate appendage is concentrically organized. The central nervous system (ependymal tube; stained pink) is centrally positioned, enclosed by the regenerated skeleton (cartilaginous cone; stained green). The skeleton is surrounding by regenerated adipose tissue (unstained) and bands of regenerated skeletal muscle (stained red). Unlike the original tail, skeletal muscle is not easily divisible into epaxial and hypaxial contributions. at, adipose tissue; bv, blood vessel; cc, cartilaginous cone; em, epaxial musculature; et, ependymal tube; hm, hypaxial musculature; nc, notochordal cartilage; ra, regenerated adipose tissue; rm, regenerated skeletal muscle; sc, spinal cord; ve, vertebra. Scale bar = 100 μm. Click here for file
