# Supplementary material for: A novel amniote model of epimorphic regeneration: the leopard gecko, Eublepharis macularius
Source: BMC Dev Biol. 2011 Aug 16;11:50. doi: 10.1186/1471-213X-11-50 (PMC3180301; doi:10.1186/1471-213X-11-50)
Supplement: Additional file 1 — Supplementary Table 1: Initial body size (snout-vent, tail, and total body length) and tail as a percentage of length for Eublepharis macularius used in this study. A list of the starting body size measurements for all 89 E. macularius taking part in this experiment. The experiment was conducted as three separate trials indicated by the specimen numbers EM 9 5 # (May 2009), EM 9 6 # (June 2009) and EM 10 2 # (February 2010). [file 1471-213X-11-50-S1.PDF]

Specimen	Snout-Vent Length (mm)	Tail Length (mm)	Total Length (mm)	Tail as a percentage of length
EM 9 5 01	90	59	149	39.6%
EM 9 5 02	103	69	172	40.1%
EM 9 5 03	85	53	138	38.4%
EM 9 5 04	95	65	160	40.6%
EM 9 5 05	90	64	154	41.6%
EM 9 5 06	81	53	134	39.6%
EM 9 5 07	81	55	136	40.4%
EM 9 5 08	82	55	137	40.1%
EM 9 5 09	97	68	165	41.2%
EM 9 5 10	94	58	152	38.2%
EM 9 5 11	100	67	167	40.1%
EM 9 5 12	90	66	156	42.3%
EM 9 5 13	101	60	161	37.3%
EM 9 5 14	93	56	149	37.6%
EM 9 5 15	96	67	163	41.1%
EM 9 5 16	95	71	166	42.8%
EM 9 5 18	89	62	151	41.1%
EM 9 5 19	98	71	169	42.0%
EM 9 5 20	90	61	151	40.4%
EM 9 5 21	74	56	130	43.1%
EM 9 5 22	86	56	142	39.4%
EM 9 5 23	84	50	134	37.3%
EM 9 5 24	85	50	135	37.0%
EM 9 6 01	70	54	124	43.5%
EM 9 6 02	70	50	120	41.7%
EM 9 6 03	73	56	129	43.4%
EM 9 6 04	74	54	128	42.2%
EM 9 6 05	73	49	122	40.2%
EM 9 6 06	76	56	132	42.4%
EM 9 6 07	68	51	119	42.9%
EM 9 6 08	69	51	120	42.5%
EM 9 6 09	72	56	128	43.8%
EM 9 6 10	70	50	120	41.7%
EM 9 6 11	75	52	127	40.9%
EM 9 6 12	73	49	122	40.2%
EM 9 6 13	72	48	120	40.0%
EM 9 6 14	71	49	120	40.8%
EM 9 6 15	72	48	120	40.0%
EM 9 6 16	70	50	120	41.7%

EM 9 6 17	72	54	126	42.9%
EM 9 6 18	72	54	126	42.9%
EM 9 6 19	74	53	127	41.7%
EM 9 6 20	77	54	131	41.2%
EM 9 6 21	72	55	127	43.3%
EM 9 6 22	73	51	124	41.1%
EM 9 6 23	70	49	119	41.2%
EM 9 6 24	71	49	120	40.8%
EM 9 6 25	72	59	131	45.0%
EM 9 6 26	74	52	126	41.3%
EM 9 6 27	75	54	129	41.9%
EM 9 6 28	75	50	125	40.0%
EM 9 6 29	71	46	117	39.3%
EM 9 6 30	73	49	122	40.2%
EM 9 6 31	77	52	129	40.3%
EM 9 6 32	75	53	128	41.4%
EM 9 6 33	66	50	116	43.1%
EM 9 6 34	76	53	129	41.1%
EM 9 6 35	77	51	128	39.8%
EM 9 6 36	72	44	116	37.9%
EM 9 6 37	72	51	123	41.5%
EM 9 6 38	70	44	114	38.6%
EM 9 6 39	72	54	126	42.9%
EM 9 6 40	74	52	126	41.3%
EM 9 6 41	72	50	122	41.0%
EM 10 2 01	63	40	103	38.8%
EM 10 2 02	53	35	88	39.8%
EM 10 2 03	54	40	94	42.6%
EM 10 2 04	62	42	104	40.4%
EM 10 2 05	56	37	93	39.8%
EM 10 2 06	60	40	100	40.0%
EM 10 2 07	54	34	88	38.6%
EM 10 2 08	58	37	95	38.9%
EM 10 2 09	57	45	102	44.1%
EM 10 2 10	57	42	99	42.4%
EM 10 2 11	55	41	96	42.7%
EM 10 2 12	56	40	96	41.7%
EM 10 2 13	58	34	92	37.0%
EM 10 2 14	55	39	94	41.5%
EM 10 2 15	60	40	100	40.0%
EM 10 2 16	60	40	100	40.0%
EM 10 2 17	60	44	104	42.3%
EM 10 2 18	60	41	101	40.6%

EM 10 2 19	60	40	100	40.0%
EM 10 2 20	58	41	99	41.4%
EM 10 2 21	60	43	103	41.7%
EM 10 2 22	56	40	96	41.7%
EM 10 2 23	55	39	94	41.5%
EM 10 2 24	59	41	100	41.0%
EM 10 2 25	60	43	103	41.7%
**Average**	**73**	**50.5**	**123.5**	**41%**
