# Supplementary figures and images for: A novel amniote model of epimorphic regeneration: the leopard gecko, Eublepharis macularius
Source: BMC Dev Biol. 2011 Aug 16;11:50. doi: 10.1186/1471-213X-11-50 (PMC3180301; doi:10.1186/1471-213X-11-50)

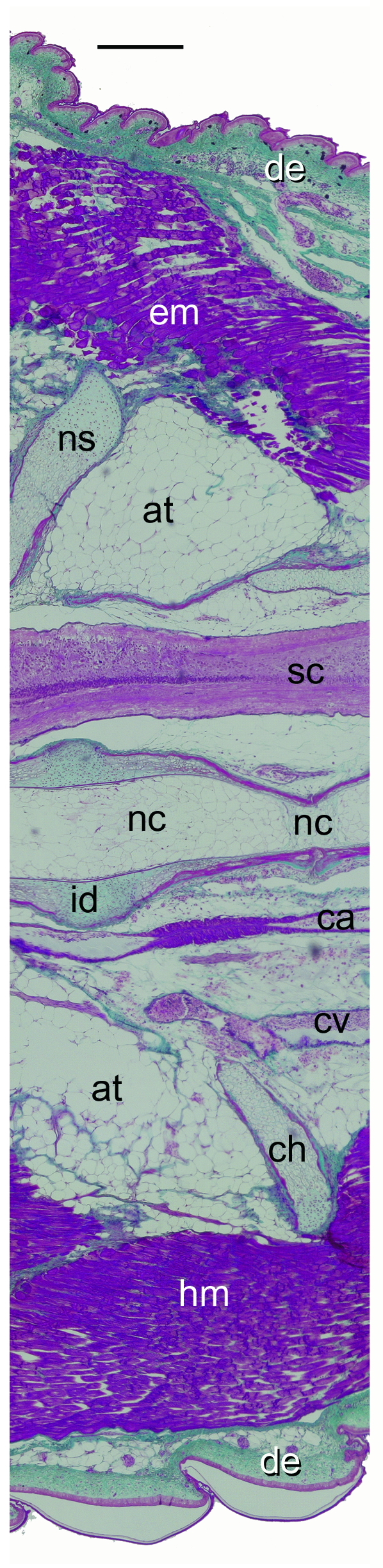

Supplement: Additional file 2 — Supplementary Figure 1: Original anatomy of the gecko tail in sagittal section. Eublepharis macularius. Serial section stained with Masson's trichrome (dorsal towards the top of the page; distal towards the left of the page). The spinal cord (stained pink) is centrally positioned. Ventral and parallel to the spinal cord is the notochord. Ventral to the vertebral column is the caudal artery (stained red). Vertebrae are surrounded by bands of adipose tissue (unstained) and skeletal muscle (stained red). The integument is composed of a thick layer of dermis (stained green) encircled by the epidermis (stained red). at, adipose tissue; ca, caudal artery; ch, chevron; cv, caudal vein; de, dermis; em, epaxial musculature; hm, hypaxial musculature; id, intervertebral disc; nc, notochordal cartilage; no, notochord; ns, neural spine; sc, spinal cord. Scale bar = 500 μm. [file 1471-213X-11-50-S2.TIFF]

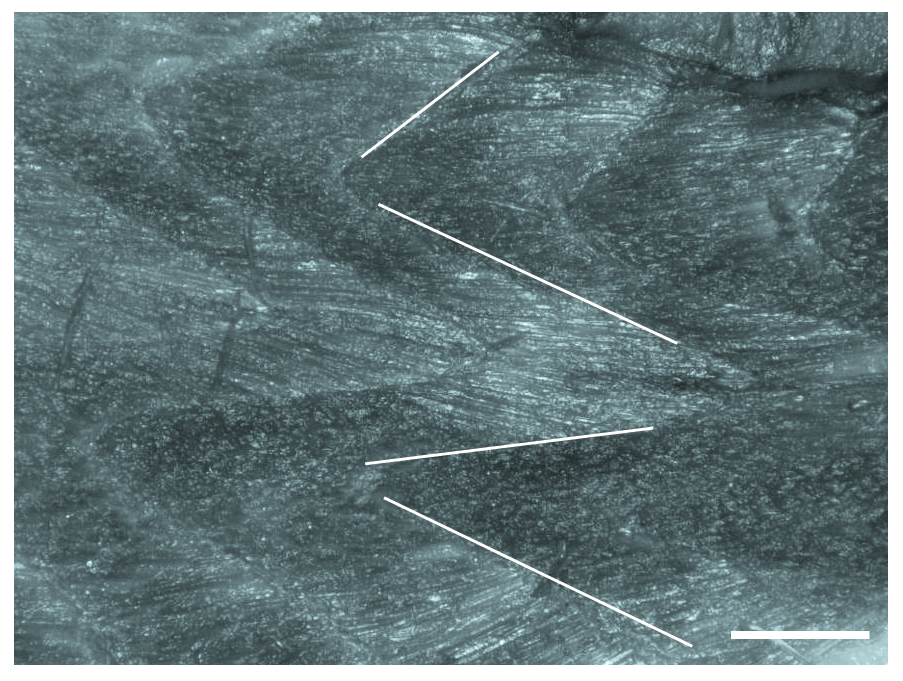

Supplement: Additional file 3 — Supplementary Figure 2: Myomere organization of the original tail. Eublepharis macularius. Gross specimen visualized with two drops of commercial red food colouring (Allura red and Erythrosine B) (dorsal towards the top of the page; distal towards the left of the page). Skeletal muscles in the tail are arranged into a prominent series of W-shaped zigzag interdigitations (white lines). Scale bar = 2 mm. [file 1471-213X-11-50-S3.TIFF]

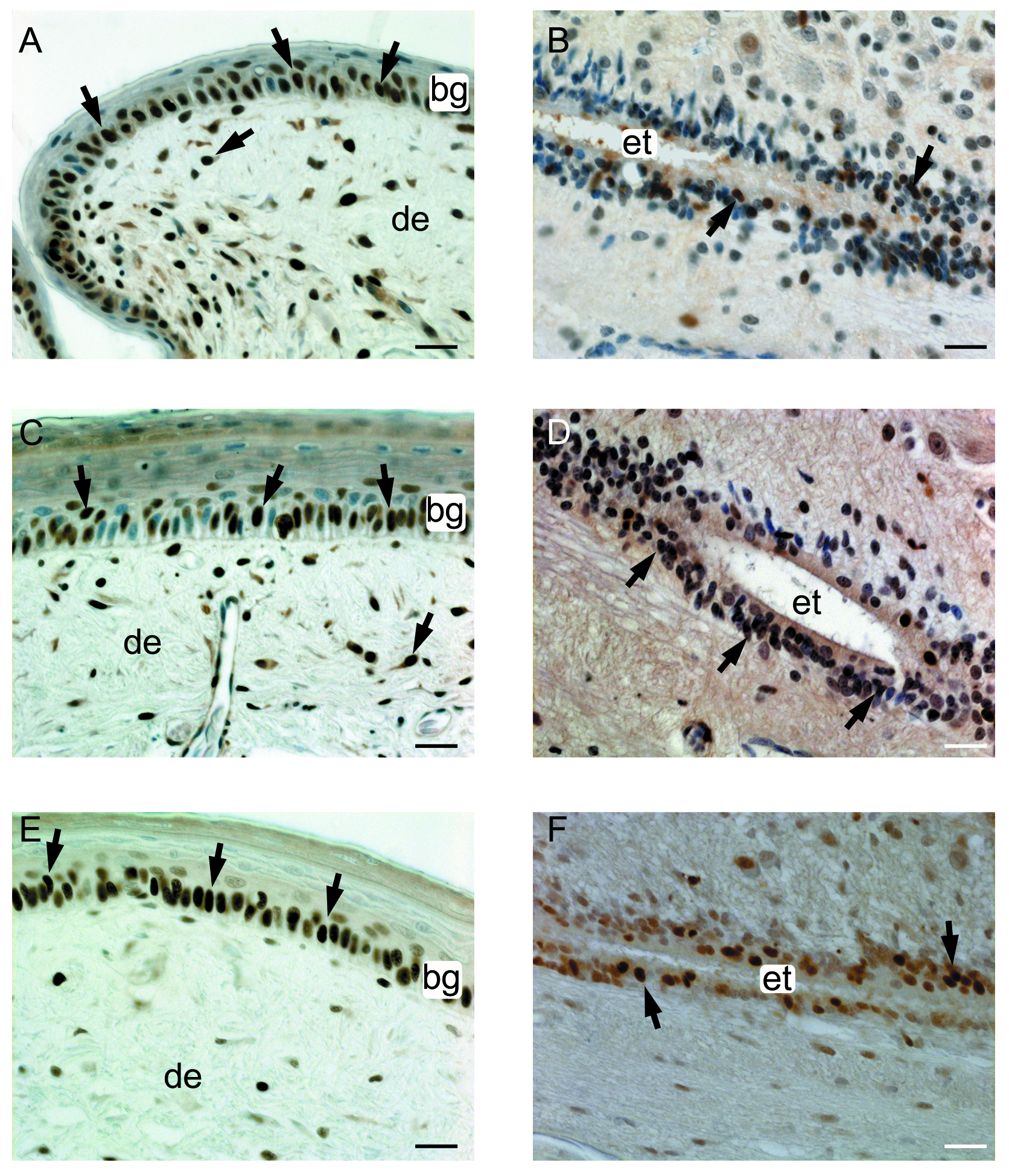

Supplement: Additional file 4 — Supplementary Figure 3: Cell proliferation in the original tail at stages II, III and IV. Eublepharis macularius. (A-D) Longitudinal sections immuostained with PCNA (brown; indicated with black arrows) and counterstained with hematoxylin (blue). (A,B) Stage II. (A) Basal germinative cells of the epidermis. (B) Ependymal cells of the ependymal tube. (C,D) Stage III. (C) Basal germinative cells of the epidermis. (D) Ependymal cells of the ependymal tube. (E,F) Stage IV. (E) Basal germinative cells of the epidermis. (F) Ependymal cells of the ependymal tube. bg, basal germinative cell; de, dermis; et, ependymal tube. Scale bar = 20 μm. [file 1471-213X-11-50-S4.TIFF]

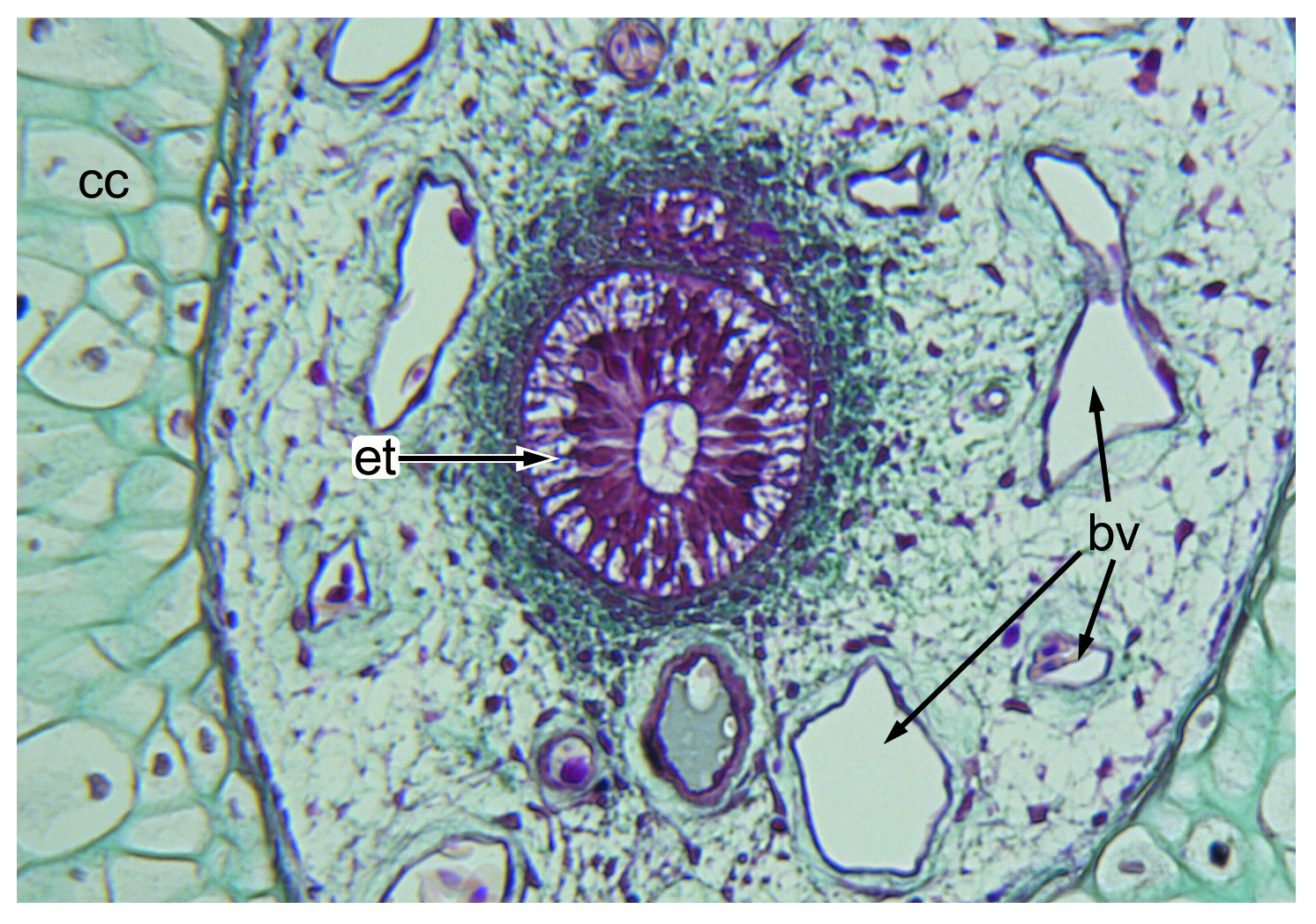

Supplement: Additional file 5 — Supplementary Figure 4: Regenerated ependymal tube. Eublepharis macularius. Transverse serial section stained with Masson's trichrome (dorsal towards the top of the page). The ependymal tube (stained pink) is centrally positioned, enclosed within the regenerated cartilaginous cone (stained green). Also within the cartilaginous cone are various blood vessels. bv, blood vessel; cc, cartilaginous cone; et, ependymal tube. [file 1471-213X-11-50-S5.TIFF]

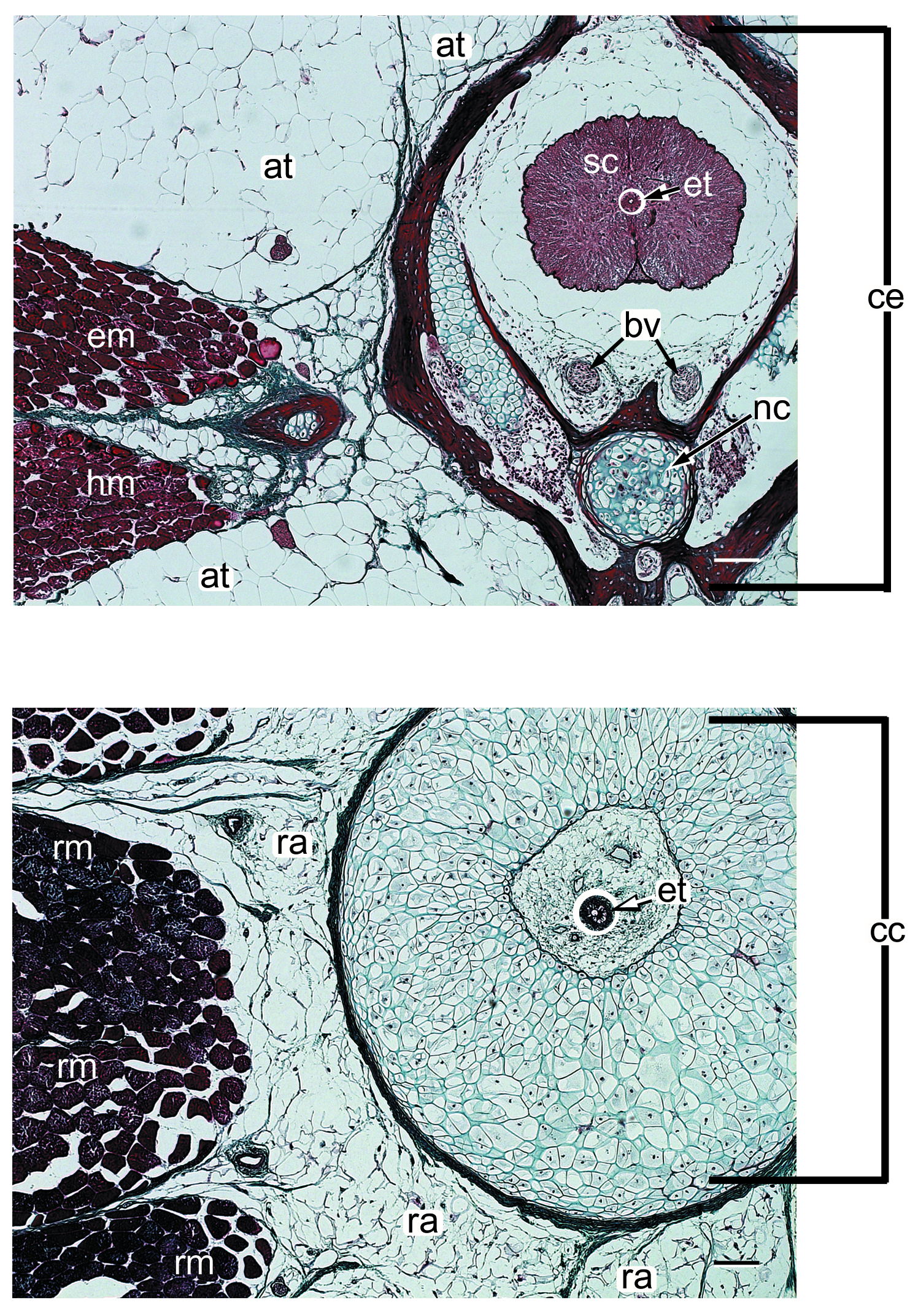

Supplement: Additional file 6 — Supplementary Figure 5: Comparison of original and fully regenerated gecko tails in transverse section. Eublepharis macularius. Serial sections stained with Masson's trichrome (dorsal towards the top of the page). (A) Original tail. The tail is concentrically organized, with the spinal cord (stained pink) enclosed within the neural canal of a vertebra (in this view stained red with green mottles). Ventral to the spinal cord is the notochord. The vertebra is enclosed by longitudinal bands of perivertebral adipose tissue, and then skeletal muscle (stained red). (B) Stage VII regenerated tail. Similar to the original tail, the regenerate appendage is concentrically organized. The central nervous system (ependymal tube; stained pink) is centrally positioned, enclosed by the regenerated skeleton (cartilaginous cone; stained green). The skeleton is surrounding by regenerated adipose tissue (unstained) and bands of regenerated skeletal muscle (stained red). Unlike the original tail, skeletal muscle is not easily divisible into epaxial and hypaxial contributions. at, adipose tissue; bv, blood vessel; cc, cartilaginous cone; em, epaxial musculature; et, ependymal tube; hm, hypaxial musculature; nc, notochordal cartilage; ra, regenerated adipose tissue; rm, regenerated skeletal muscle; sc, spinal cord; ve, vertebra. Scale bar = 100 μm. [file 1471-213X-11-50-S6.TIFF]
